# A genome-engineered tool set for *Drosophila* TGF-β/BMP signaling studies

**DOI:** 10.1242/dev.204222

**Published:** 2024-11-18

**Authors:** Clara-Maria Ell, Abu Safyan, Mrinal Chayengia, Manuela M. M. Kustermann, Jennifer Lorenz, Melanie Schächtle, George Pyrowolakis

**Affiliations:** ^1^ Spemann Graduate School of Biology and Medicine (SGBM), University of Freiburg, 79104 Freiburg, Germany; ^2^ CIBSS – Centre for Integrative Biological Signalling Studies, University of Freiburg, 79104 Freiburg, Germany; ^3^ Institute for Biology I, Faculty of Biology, HMH, Habsburgerstr. 49, University of Freiburg, 79104 Freiburg, Germany; ^4^ International Max Planck Research School for Immunobiology, Epigenetics, and Metabolism, 79108 Freiburg, Germany

**Keywords:** TGF-β/BMP signaling, Functionalized protein binder tools, Wing imaginal disc, *Drosophila* development, Endogenous protein tagging, Oogenesis

## Abstract

Ligands of the TGF-β/BMP superfamily are crucially involved in the regulation of growth, patterning and organogenesis and can act as long-range morphogens. Essential for understanding TGF-β/BMP signaling dynamics and regulation are tools that allow monitoring and manipulating pathway components at physiological expression levels and endogenous spatiotemporal patterns. We used genome engineering to generate a comprehensive library of endogenously epitope- or fluorescent-tagged versions of receptors, co-receptors, transcription factors and key feedback regulators of the *Drosophila* BMP and Activin signaling pathways. We demonstrate that the generated alleles are biologically active and can be used for assessing tissue and subcellular distribution of the corresponding proteins. Furthermore, we show that the genomic platforms can be used for *in locus* structure-function and *cis*-regulatory analyses. Finally, we present a complementary set of protein binder-based tools, which allow visualization as well as manipulation of the stability and subcellular localization of epitope-tagged proteins, providing new tools for the analysis of BMP signaling and beyond.

## Teaser

**Summary:** A comprehensive collection of endogenously epitope- or fluorescent-tagged components of the *Drosophila* TGF-β/BMP signaling pathway can be used to assess localization and function of the corresponding proteins.

## INTRODUCTION

Transforming growth factor β (TGF-β)/Bone Morphogenetic Protein (BMP) signaling is crucial for animal development and homeostasis and is deregulated in various pathologies and diseases (Jia and Meng, 2021; Massagué and Sheppard, 2023). Despite context-dependent differences in complexity and regulation, the core pathway of canonical TGF-β/BMP signaling that transmits information from extracellular ligands to the nuclei of signal-receiving cells is relatively simple and evolutionarily highly conserved. Ligands of the TGF-β, BMP and Activin families assemble into dimers and bind to extracellular domains of membrane-bound type I and type II receptor serine-threonine kinases. Upon ligand binding, type II receptors phosphorylate a glycine- and serine-rich juxtamembrane domain (GS domain) of type I receptors. Activated type I receptors then phosphorylate C-terminal serine residues of receptor-associated Smads (R-Smads), which associate with common Smad (co-Smad) and accumulate in the nucleus. Here, the Smad complexes directly bind to DNA and regulate, together with other transcription factors and co-regulators, target gene transcription.

**Fig. 1.**
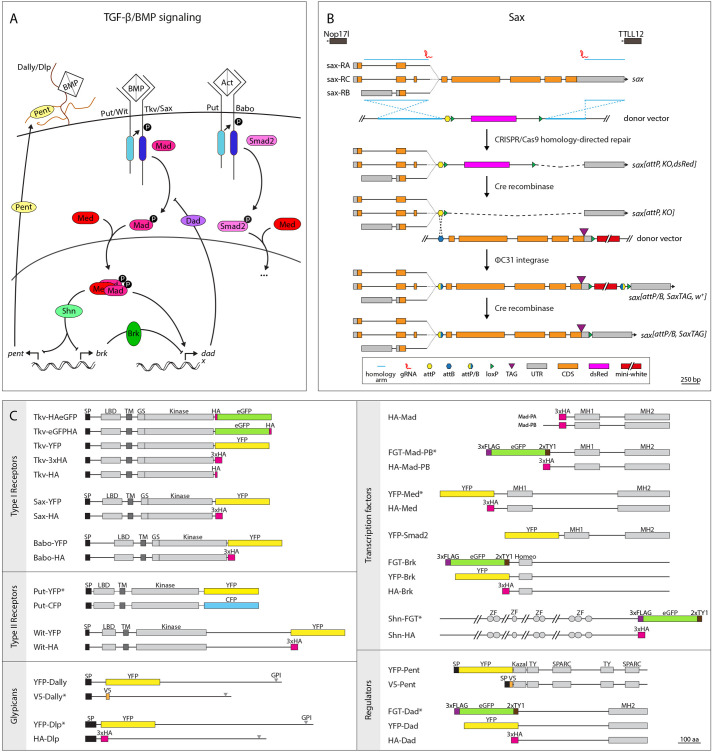
**Genome engineering *Drosophila* TGF-β/BMP signaling.** (A) Overview of the TGF-β/BMP signaling pathway, with an emphasis on components that have been modified in this study (shown in color). (B) Two-step genome-engineering strategy exemplified with Sax. CRISPR/Cas9-mediated homology-directed repair was used to replace the target sequence with an attP-containing cassette. After removal of the dsRed selection marker, the genomic locus was restored with tagged versions of the gene by standard ФC31 transgenesis. The mini-white selection marker was excised by Cre recombinase. Exact strategies for the other TGF-β/BMP components are depicted in Fig. S1. Note that, in the case of Dally, Pent, Tkv and Put, homologous recombination as described in Baena-Lopez et al. (2013) was used to generate the attP,KO lines. bp, base pairs; CDS, coding sequence; UTR, untranslated region. (C) In-scale overview of tagged TGF-β/BMP components to visualize the position of the tag (shown in color) in relation to protein domains. At least one but up to five tagged variants are available for all components. For simplicity, all components tagged with 3×HA are termed HA-X or X-HA. Note the exception of Tkv, for which the 3×HA-tagged version is termed Tkv-3×HA, a single HA-tagged version is termed Tkv-HA, and versions tagged with a single copy of HA and eGFP are termed Tkv-HAeGFP and Tkv-eGFPHA. Variants marked by an asterisk are not viable in homozygosity. Grey rectangles at the glypicans indicate the position of the glycosylphosphatidylinositol (GPI) attachment site. aa, amino acids; GPI, GPI anchor; GS, GS domain; Homeo, homeodomain; Kazal, Kazal domain; Kinase, kinase domain; LBD, ligand-binding domain; MH1, MH1 domain; MH2, MH2 domain; SPARC, Secreted Protein Acidic and Rich in Cysteine domain; SP, signal peptide; TM, transmembrane domain; TY, thyroglobulin type 1 domain; ZF, zinc finger domain.

The high evolutionary conservation of the TGF-β/BMP signaling pathway has allowed the use of model organisms such as *Drosophila* to identify many components of the pathway and to unravel key concepts of signal transduction and regulation of the pathway over the past years (Fig. 1A) (Akiyama et al., 2024). In *Drosophila*, ligands of the BMP and Activin branches of the TGF-β/BMP superfamily are required throughout fly development and homeostasis to regulate processes such as cell proliferation, cell differentiation, cell migration and apoptosis (Upadhyay et al., 2017). The best-studied BMP ligand in *Drosophila* is Decapentaplegic (Dpp), the fly homolog of vertebrate BMP2 and BMP4. Dpp, which often forms a heterodimer with Glass bottom boat (Gbb) or Screw (Scw), the other *Drosophila* BMPs, plays essential roles in embryonic dorsoventral axis formation, germline and intestinal stem cell maintenance, patterning and growth of larval imaginal discs, and patterning of the follicular epithelium during oogenesis (Akiyama et al., 2024; Upadhyay et al., 2017). Detailed studies in some of these tissues underlined the necessity of precise spatiotemporal regulation of Dpp/BMP signaling activity (Bier and De Robertis, 2015; Montanari et al., 2022; Wilcockson et al., 2017). This is particularly evident in the context of larval wing development, during which Dpp, in parallel with its role in promoting growth, acts as a morphogen to provide positional information (Affolter and Basler, 2007; Ashe and Briscoe, 2006; Hamaratoglu et al., 2014; Kicheva and Briscoe, 2023; Stapornwongkul and Vincent, 2021). Decades of research on wing development, which has widely served as a paradigm for morphogen signaling, have highlighted a number of determinants and interactions shaping the BMP activity gradient. Although localized expression of *dpp* in a stripe of anterior cells at the anterior-posterior compartment boundary and dispersion from this source are thought to underlie gradient formation, the exact mechanisms by which Dpp spreads into both compartments are not completely understood. Nevertheless, it has become evident that the sum of multiple interactions acting at distinct levels regulate gradient shape and transcriptional output. At the membrane surface, the distribution, levels and activity of the main receptor Thickveins (Tkv), the glypican Division abnormally delayed (Dally) and the Dally-binding protein Pentagone (Pent, also known as Magu) are crucially involved in the distribution and activity of Dpp (Akiyama et al., 2024). Within the cytoplasm of the cell, Daughters against dpp (Dad) serves as an inhibitory Smad (I-Smad) and feedback regulator of the pathway to regulate phosphorylation levels of the R-Smad Mothers against dpp (Mad). Finally, the output of the gradient in the nucleus, i.e. the transcriptional activation of BMP target genes, is controlled by the transcriptional repressor Brinker (Brk), which counteracts Smad-dependent gene activation and is itself directly repressed by the nuclear Smad gradient (Affolter and Basler, 2007; Hamaratoglu et al., 2014). A similarly high necessity for spatiotemporal regulation of BMP signaling activity has been illustrated, or is expected, in other developmental and homeostatic contexts, including BMP-dependent embryonic axis determination, intestinal and germline stem cell (GSC) maintenance and regeneration, synaptogenesis, as well as follicle cell patterning (Akiyama et al., 2024; Bier and De Robertis, 2015; Hamaratoglu et al., 2014; O'Connor et al., 2006; Pyrowolakis et al., 2017; Umulis et al., 2009; Wilcockson et al., 2017).

Analyses of BMP signaling in the above-mentioned contexts require reagents and tools that allow for monitoring and manipulating the involved proteins at physiological levels and endogenous expression patterns. We present here a collection of genome-engineered core components of the pathway that allows efficient epitope and fluorescent protein tagging, and visualization of the tissue and cellular distribution of the corresponding proteins. Furthermore, we show how the genomic platforms can be used to address isoform and structural requirements for protein function. To this end, we demonstrate that a hitherto uncharacterized Mad isoform, Mad-PB, is positively regulated by BMP signaling in the wing disc and is essential for proper adult wing size. In addition, we show that membrane anchoring, but not necessarily glycosylphosphatidylinositol (GPI)-mediated anchoring, of the glypican Dally is required for its biological activity. Lastly, we present a complementary set of tools based on a protein binder against the HA epitope tag, which, along with previously established nanobody-based tools against GFP, can be used for trapping, mislocalizing or degrading tagged proteins from our library.

## RESULTS

### Generation of endogenously tagged TGF-β/BMP components

We employed a two-step protocol to generate endogenously epitope- or fluorescent-tagged TGF-β/BMP signaling components (Fig. 1; Fig. S1). In the first step, we used genome engineering to replace a selected region of the target gene with a sequence containing an attP site along with a loxP-flanked selection cassette. The exact strategy and the choice of the introduced deletion were individually adapted to account for the genomic constraints and architecture of the respective target locus as well as the domain structure of the corresponding protein (Fig. 1B; Fig. S1A). Overall, we generated chromosomal lesions in 14 genes of the *Drosophila* TGF-β/BMP signaling pathway. The introduced deletions were verified both genetically and molecularly (see Materials and Methods and Fig. S1B,C) and constitute a collection of new, molecularly defined null alleles of the corresponding genes. In the second step, we used ФC31/attB integration to reinstate the genomic locus with epitope- or fluorescent-tagged versions of the gene. Depending on the target gene and the introduced deletion, the strategy varied to reintegrate single exons, full-length cDNA versions of the gene, or extended genomic sequences including introns and/or flanking intergenic sequences. We introduced a number of tags including eGFP, YFP, CFP, HA (in single or triple copies), V5 and FGT (a fusion of 3×FLAG, eGFP and 2×TY1; described by Sarov et al., 2016). Depending on the protein, tags were introduced either at the C-terminus (receptors) or at the N-terminus (most transcription factors) (Fig. 1C). For proteins that contain signal peptides and do not tolerate C-terminal modifications (Pent and glypicans), tags were introduced internally, close to the N-terminus but downstream of the signal peptidase cleavage site. The modified genes were tested for their ability to substitute for the function of the corresponding endogenous gene. At least one (and up to five) of our epitope- or fluorescent-tagged versions of each gene is functional, based on the criterion that flies carrying the introduced modifications in homozygosity were viable and fertile, and did not display gross phenotypic abnormalities (Fig. 1C). All modified components have been reported to contribute to wing development, a tissue that is particularly sensitive to perturbations of TGF-β/BMP signaling. To address the biological activity of the tagged proteins, we scored for morphological abnormalities in adult wings that carried the modified components in homozygosity (Fig. S2). In the majority of the cases, wings were normal in size and morphology, indicating that the modified protein was fully functional. Notable exceptions were the wings of flies homozygous for the gene encoding Tkv-YFP, which occasionally displayed venation defects including truncations at the distal tip and local thickening of veins. As these phenotypes were only present in flies carrying Tkv-YFP but not in flies carrying the other modified versions of Tkv, we conclude that they are not caused by the tagging approach or the position of the tag, but rather by the nature of the tag.

### Assessing tissue and cellular distribution of membrane-bound TGF-β/BMP components

**Fig. 2.**
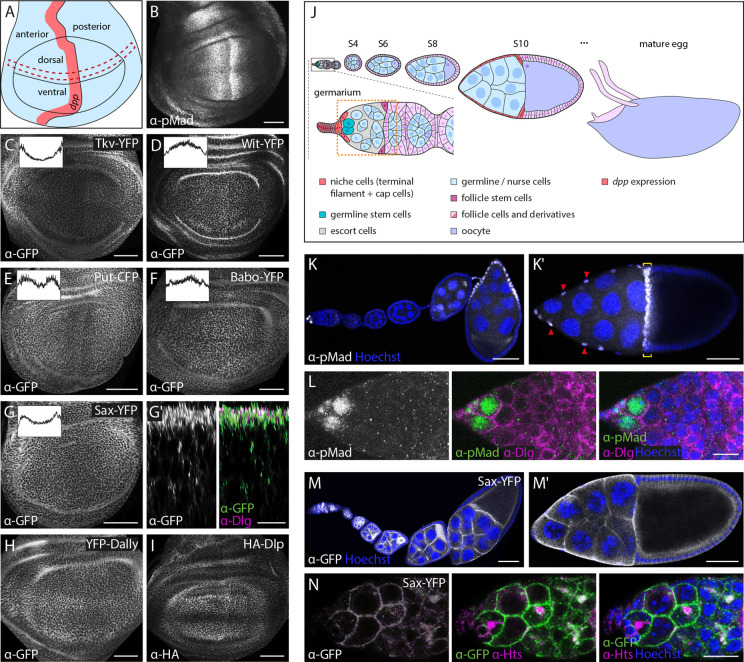
**Tissue and cellular distribution of tagged TGF-β/BMP receptors and glypicans.** (A) Schematic depiction of the larval wing imaginal disc showing the *dpp* expression domain in the anterior compartment in red. Red dashed box indicates area used to generate intensity plots shown in C-G. (B) Anti-pMad staining shows the BMP signaling activity gradient in the wing imaginal disc. (C-I) Anti-GFP or anti-HA staining visualizes the distribution of receptors (C-G) and glypicans (H,I) in wing imaginal discs. Insets show intensity plots along the anterior-posterior disc axis. (G′) Subcellular localization of Sax-YFP in the disc in relation to the septate junction marker Discs large (Dlg) (magenta). (J) Schematic depiction of selected stages of oogenesis. Cells expressing *dpp* (niche cells in the germarium, stretched follicle cells in S10) are shown in red. The orange dashed box indicates area of the germarium shown in panels L,N and in Fig. S5. S, stage. (K,Kʹ,L) Anti-pMad staining visualizes BMP signaling activity during oogenesis. During egg chamber development, pMad is present in anterior most follicle cells and, at S10, in both nurse cell-associated stretched follicle cells (red arrowheads, K′) and a band of anterior oocyte-associated follicle cells (yellow brackets, K′). In the germarium, pMad levels are high in germline stem cells (L). Nuclei are stained by Hoechst (blue) and anti-Dlg staining is shown in magenta. (M,Mʹ,N) Anti-GFP staining of Sax-YFP shows its distribution during oogenesis. Nuclei are visualized by Hoechst (blue) and spectrosomes by anti-Hu li tai shao (Hts) staining (magenta). Images are representative of at least five wing discs or ovaries. Scale bars: 50 μm (B-I,K,M); 10 μm (Gʹ,L,N).

**Fig. 3.**
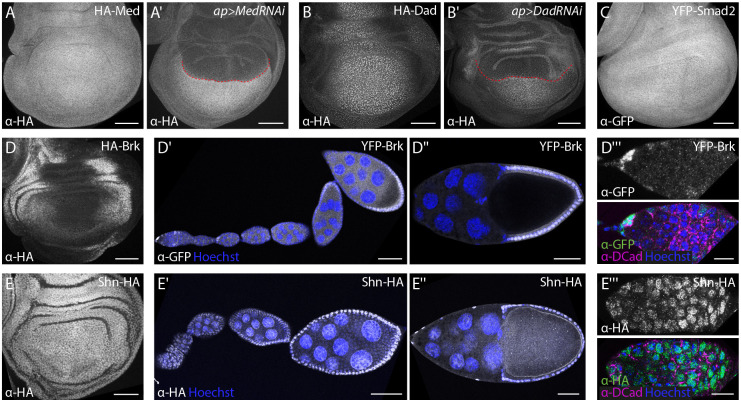
**Tissue distribution of intracellular TGF-β/BMP components.** (A-E) Anti-GFP or anti-HA staining visualizes the distribution of tagged Smad proteins (A-C) and transcription factors (D,E) in wing imaginal discs. (A′,B′) RNAi-mediated depletion of Med (A′) and Dad (B′) in the dorsal wing disc compartment using ap-Gal4 verified the specificity of the observed staining. The red dashed line indicates the dorsoventral compartment boundary. (D′-E‴) Anti-GFP and HA-staining of YFP-Brk (D′-D‴) and Shn-HA (E′-E‴), respectively, show their distribution during oogenesis. Nuclei are visualized by Hoechst (blue) and anti-DE-Cadherin (DCad) staining is shown in magenta. Images are representative of at least five wing discs or ovaries. Scale bars: 50 μm (A-E″); 10 μm (D‴,E‴).

**Fig. 4.**
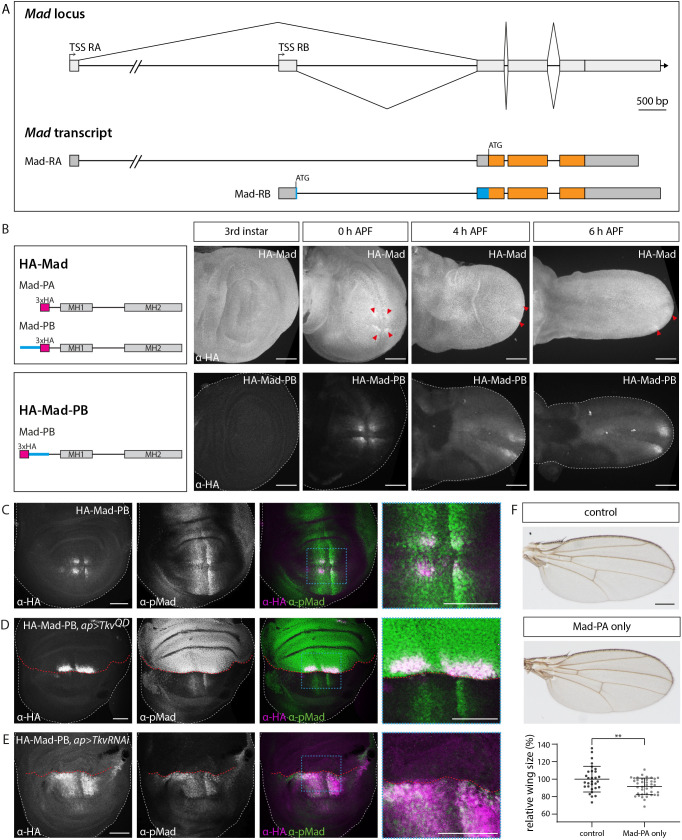
***Mad* isoform analysis.** (A) *Mad* genomic locus and the predicted *Mad* transcript isoforms. *Mad-RB* contains an N-terminal extension (highlighted in blue). Orange boxes mark coding sequences and grey boxes mark untranslated regions. bp, base pairs; TSS, transcriptional start site. (B) Anti-HA staining of endogenously tagged Mad versions visualized in third instar wing discs and early prepupal wings. HA-Mad tags both isoforms, whereas HA-Mad-PB exclusively tags Mad-PB. Discs are oriented with the anterior up and posterior down. Red arrowheads indicate patches of elevated protein levels, possibly corresponding to HA-Mad-PB in addition to the uniform HA-Mad-PA expression. White dashed lines indicate disc outlines. APF, after puparium formation. Images are representative of at least five larval or prepupal wings. (C-E) Anti-HA and anti-pMad immunostaining in late larval wild-type wing discs (C) and discs expressing Tkv^QD^ (D) or Tkv-RNAi (E) in the dorsal compartment using ap-Gal4. Discs are oriented with the anterior to the left and posterior to the right. White dashed lines indicate disc outlines, and red dashed lines indicate the dorsoventral compartment boundary. Panels on the right are magnifications of the areas indicated by the blue dashed boxes. Images are representative of at least five wing discs. (F) Adult wings of control flies (carrying a reintegration of the wild-type genomic *Mad* sequence in *mad^[attP, KO]^*) and flies that only contain Mad-PA. The graph shows relative wing size (as a percentage) of the indicated genotypes, shown as mean±s.d. Dots represent the measured size of individual wings isolated from male flies (control, *n*=30; Mad-PA only, *n*=40). Statistical significance was analyzed by a two-tailed unpaired *t*-test with Welch's correction assuming unequal variances. **P≤0.01. Scale bars: 50 μm (B-E); 250 μm (F).

With the above collection at hand, we tested whether our endogenously modified alleles can be used to monitor the distribution of the proteins in tissues and cells. We focused on three tissues, which crucially depend on TGF-β/BMP signaling and its tight spatiotemporal regulation for proper development and function: the larval wing imaginal discs, the ovarian germarium and the developing egg chamber (Pyrowolakis et al., 2017; Upadhyay et al., 2017; Wilcockson et al., 2017) (see Fig. 2A,B,J-L for BMP activity patterns in the three settings). In the wing imaginal disc, Activin signals provide growth cues and BMP signals (ligands Dpp and Gbb) provide growth and patterning cues, with the latter acting as a morphogen. There is ample genetic and direct evidence that all modified genes of our library are expressed during larval wing development and are involved in the transmission and regulation of TGF-β/BMP signals. Indeed, we found robust expression of all tested components in the larval wing imaginal disc (Figs 2, 3 and 4). Receptors (Tkv, Put, Sax, Babo and Wit) and glypicans (Dally and Dlp) could be readily visualized by immunostaining against the YFP and/or HA tags (Fig. 2C-I) and depletion of the corresponding transcripts by gene-specific RNAi resulted in a loss of signal, confirming specificity (Fig. S3). Our analyses indicate that most, if not all, receptors and glypicans display patterned distribution rather than being uniformly present in the wing disc epithelium. For the receptor Tkv, non-uniform expression along the anterior-posterior axis of the wing disc has been described and this depends on both BMP and Hedgehog (Hh) signaling pathways that repress transcription of *tkv* in medial cells of the disc (Crickmore and Mann, 2006; Lecuit and Cohen, 1998; Tanimoto et al., 2000). The distribution of our Tkv variants was consistent with these observations (Fig. 2C). In addition, recent work has shown that the expression of Wit (a BMP type II receptor) is transcriptionally activated by BMP signaling during larval wing development (Chayengia et al., 2019). These findings were recapitulated in the distribution of Wit-YFP, which was increased in the medial and lower in lateral cells of the wing pouch (Fig. 2D). Put, the essential type II receptor for BMP signaling (Letsou et al., 1995; Ruberte et al., 1995), also displayed modulated distribution along the anterior-posterior axis of the disc, with lower levels at the positions of the two peaks of phosphorylated Mad (pMad) on either side of the Dpp source (Fig. 2E). Babo, a type I receptor for Activin controlling larval wing size (Brummel et al., 1999), displayed increased expression in medial cells (Fig. 2F). Lastly, Sax, a type I receptor with a crucial contribution to long-range BMP signaling (Bangi and Wharton, 2006; Brummel et al., 1994; Haerry et al., 1998; Nellen et al., 1994; Nguyen et al., 1998; Penton et al., 1994; Xie et al., 1994), displayed increased expression in lateral regions of the disc (Fig. 2G).

The glypicans Dally and Dlp have been demonstrated to be modulated in their expression along the anterior-posterior or dorsoventral axes of the wing and to differentially affect the activities of Wingless (Wg), Dpp, Hedgehog (Hh) and other signaling pathways during wing development (Baeg et al., 2001; Belenkaya et al., 2004; Franch-Marro et al., 2005; Fujise et al., 2003; Han et al., 2004, 2005; Kirkpatrick et al., 2004; Kreuger et al., 2004; McGough et al., 2020; Simon et al., 2021, 2024). The complex expression pattern of Dally has been mainly deduced from enhancer trap lines and was shown to contribute to BMP gradient formation (Crickmore and Mann, 2007; de Navas et al., 2006; Fujise et al., 2001, 2003; Makhijani et al., 2007). Broadly in line with these reports, our YFP-Dally construct displayed increased protein levels in lateral regions and along the dorsoventral compartment boundary of the wing disc while showing low expression in medial regions (Fig. 2H). However, our allele failed to reflect the prominent peak of *dally* expression in the *dpp*-expressing stripe reported by *dally-lacZ* traps (Fujise et al., 2003). HA-Dlp, in agreement with previous reports (Han et al., 2005), was expressed at high levels in the dorsal and ventral cells of the pouch and was particularly low in a broad stripe of cells straddling the dorsoventral compartment boundary due to Wg-mediated repression (Fig. 2I).

We also addressed the distribution of the fusion proteins along the apical-basal axis of the polarized cells of the wing (Fig. 2G′; Fig. S4). Apical and basolateral pools were detectable for all of the receptors and glypicans with the exception of Put, which appeared to be excluded from apical cell membranes, consistent with recent reports using UAS-based and rescue constructs of the gene (Peterson et al., 2022).

We observed robust but variegated expression of the tagged components during oogenesis (Fig. 2M,N; Fig. S5). Sax expression, for example, was mostly absent in follicle cells but present in the germline, with signals detectable already in GSCs and persisting in nurse cells and oocytes throughout oogenesis (Fig. 2M,M′,N). This distribution is consistent with the requirements of Sax in the germline during oogenesis and egg chamber formation (Twombly et al., 1996; Xie and Spradling, 1998). Other receptors, including Tkv, Babo and Put, were expressed in both somatic and germline cells, although at distinct spatial patterns and stages (Fig. S5A-F). Genetic studies have firmly established that Tkv transduces BMP signals in GSCs for stem cell maintenance and is also involved in the control of ligand distribution by patterned expression and expression in somatic cells of the ovarian niche (Luo et al., 2015; Ma and Xie, 2011; Michel et al., 2011; Tseng et al., 2018; Wilcockson and Ashe, 2019; Xia et al., 2012). Consistently, we observed widespread presence of Tkv in the germarium, with increased levels in the posterior-most cell population (Fig. S5B). In addition, Tkv was strongly expressed in the oocyte, where it displayed a diffuse cytosolic distribution at early stages and a more juxtamembrane localization at later stages of oogenesis (Fig. S5A,A′). Moreover, and in agreement with its role in eggshell patterning (Yakoby et al., 2008), Tkv was found at membranes of follicle cells throughout oogenesis (Fig. S5A,A′). Babo, which has been genetically implicated in mediating Activin signaling in ovarian niche development (Lengil et al., 2015), was widely expressed in the germarium (but not in germline cells at later stages) and in follicle cells throughout oogenesis (Fig. S5C,C′,D), in which no role of Activin/Babo signaling has been described so far. Put, which is genetically required for GSC maintenance (Kawase et al., 2004), was present at membranes of germline cells throughout oogenesis and was also robustly expressed in follicle cells (Fig. S5E,E′,F).

Both the *Drosophila* glypicans Dally and Dlp have been functionally implicated in GSC niche homeostasis (Guo and Wang, 2009; Hayashi et al., 2009). Dally restricts BMP signaling to the anterior cells of the niche and is exclusively expressed in cap and terminal filament cells as judged by enhancer trap lines (Guo and Wang, 2009; Hayashi et al., 2009; Liu et al., 2010). Our tagged Dally allele, which fully restored fertility of *dally^[attP,KO]^* mutants, could be detected at low levels on the membranes of somatic cells of the germarium (Fig. S5G,H). The discrepancy with the strong reporter expression in *dally-lacZ* flies might stem from low expression levels or limited stability of the protein compared to that of the stable β-galactosidase in the cells of the niche. Indeed, at later stages of oogenesis, the same allele was readily detectable at membranes of developing follicle cells throughout oogenesis (Fig. S5G,G′), consistent with previous reports (Su et al., 2018). In contrast, Dlp was absent in the follicular epithelium at all stages of egg chamber development but clearly expressed in cells of the germarium (Fig. S5I,I′,J). The distribution of Dlp in the germarium with lower levels in escort cells (inner germariar cells) is in line with recent studies describing Dlp expression in the germarium and its role in regulating GSC differentiation (Tu et al., 2020; Waghmare et al., 2020).

### Assessing tissue and cellular distribution of intracellular TGF-β/BMP components

Next, we assessed the distribution of intracellular components of the TGF-β/BMP signaling pathway in the same contexts (Fig. 3). The *Drosophila* Smads Med and Smad2 (also known as Smox) (for Mad, see the next section) were expressed at low and uniform levels in the wing imaginal disc, with no signs of nuclear enrichment (Fig. 3A,C). Dad, the single inhibitory Smad in *Drosophila*, was distributed in a pattern that is consistent with the well-established Dpp-dependent transcriptional regulation of the gene (Fig. 3B) (Tsuneizumi et al., 1997; Weiss et al., 2010): *dad* transcription is directly activated by Smad complexes in medial cells of the disc and repressed by Brk in lateral cells. All signals were specific as they were lost upon RNAi-mediated depletion of the fusion proteins in the dorsal compartment (Fig. 3A′,B′). Tagged versions of Brk were distributed in a complementary nuclear gradient to the distribution of Dad, with highest levels in lateral cells and declining levels towards medial regions of the wing disc (Fig. 3D) (Marty et al., 2000; Müller et al., 2003; Pyrowolakis et al., 2004; Yao et al., 2008). Similarly, and in agreement with previous studies based on RNA *in situ* hybridization and reporter analyses (Charbonnier et al., 2015; Chen and Schüpbach, 2006; Shravage et al., 2007), the expression of tagged Brk in the follicular epithelium was restricted to posterior follicle cells that are devoid of BMP signaling activity (Fig. 3D′,D″). In both epithelia, *brk* expression is directly repressed by BMP signaling through Smad-dependent recruitment of Shn to *cis*-regulatory modules of the gene (Charbonnier et al., 2015; Marty et al., 2000; Müller et al., 2003; Pyrowolakis et al., 2004; Torres-Vazquez et al., 2000; Yao et al., 2008). Accordingly, Shn displayed a uniform, nuclear expression pattern in both the wing disc and the follicular epithelium (Fig. 3E-E″). In addition, Shn was present in the germarium and especially in the nuclei of germline cells (Fig. 3E‴), suggesting a role in BMP signaling-mediated GSC maintenance, which parallels findings from the male germline (Matunis et al., 1997). Finally, Brk was weakly present in the germline but strongly expressed in somatic cells at the tip of the niche (Fig. 3D‴), consistent with a recent report on the role of Brk in regulating *dpp* expression in cap cells (Dunipace et al., 2022).

### An autoregulatory loop in *Mad* isoform expression

Mad is the founding member of the Smad transcription factor family and is the only BMP-responsive Smad encoded in the *Drosophila* genome. The ModEncode project (Graveley et al., 2011) predicts the existence of two *Mad* transcript isoforms (referred to as *Mad-RA* and *Mad-RB*, respectively; see http://flybase.org/reports/FBgn0011648) arising by the use of alternative transcriptional start sites (Fig. 4A; Fig. S6). To our knowledge, *Mad-RA* (and the corresponding protein Mad-PA) is the isoform considered and exclusively used in previous studies that address Mad function, mostly through UAS-based assays or cell culture expression. Mad-PB, the product of the *Mad-RB* transcript, bears an N-terminal extension of 70 amino acids that contains no discernable motifs and is of generally low complexity (Fig. 4A,B; Fig. S6F). Based on developmental expression profiling data, *Mad-RA* transcripts are robustly detectable throughout development, whereas expression of the *Mad-RB*-specific exon can be detected during embryogenesis (but it is not maternally provided), declines during larval development, and reappears at late larval stages and pupal development (Fig. S6A). We used our attP platform introduced into the *Mad* locus to address both the expression and function of the potential isoforms. We inserted three copies of the HA sequence directly after the ATG codons of either isoform to monitor protein distribution in wing imaginal discs. Note that, as Mad-PB represents an N-terminal, in-frame extension of Mad-PA, genomic insertion of the tag at the start codon of *Mad-RA* would theoretically result in an N-terminally HA-tagged Mad-PA and a Mad-PB version that carries an internal tag at amino acid position 71 (Fig. 4B; Fig. S6F). We refer to this construct as HA-Mad to indicate that both potential isoforms carry the HA tag. In contrast, insertion of the HA tag at the start codon of *Mad-RB* should exclusively label the Mad-PB polypeptide at its N-terminus; we refer to this allele as HA-Mad-PB. Larval wings at third instar stages displayed a ubiquitous HA-Mad distribution, and some additional distinct patches of slightly increased levels in regions flanking the Dpp source cells were visible in prepupal stages (Fig. 4B; Fig. S6B). Consistent with early reports using antibodies against Mad (Newfeld et al., 1997), the distribution of HA-Mad was uniform with no signs of nuclear enrichments in regions of high BMP signaling activity, suggesting that only a minor fraction of the protein accumulates in the nucleus upon phosphorylation. At the same time, HA-Mad-PB was absent in third instar wing discs until very late larval stages, where it appeared in four patches arranged around the intersection point of the compartment boundaries, which persisted and expanded during early pupal development (Fig. 4B; Fig. S6C,D). Notably, the patches of HA-Mad-PB coincided with peak levels of pMad in both the anterior and the posterior compartment (Fig. 4C), suggesting that BMP signaling is involved in the regulation of Mad expression. Indeed, increasing BMP activity by overexpression of a constitutively active Tkv receptor in the dorsal compartment resulted in an expansion of the two dorsal patches of HA-Mad-PB without causing premature expression (Fig. 4D; Fig. S6E). Note that this manipulation also resulted in a drastic reduction of pMad and HA-Mad-PB in ventral cells. In reverse, RNAi-induced depletion of Tkv resulted in a loss of HA-Mad-PB expression in dorsal cells (Fig. 4E). At the same time, both the pMad gradient and HA-Mad-PB levels increased in ventral cells, with the effect being stronger in cells abutting the dorsoventral compartment boundary. The non-autonomous effects in ventral cells most probably reflect redistribution of BMP ligands due to the increase or decrease of receptor levels in the dorsal compartment as reported before (for example, see Crickmore and Mann, 2006; Simon et al., 2024). Cumulatively, the results demonstrate that BMP signaling positively regulates Mad-PB expression.

To understand the relative contribution of the two isoforms in fly BMP signaling, we introduced mutations in the genomic sequence of *Mad* to selectively express Mad-PA or Mad-PB (Fig. S6G). We refrained from inserting epitope tags into these constructs to avoid confounding effects from such elements in the protein sequence. As a control, we also reintegrated the wild-type genomic *Mad* sequence using the same strategy in the *mad^[attP,KO]^* site. Flies carrying the control genomic sequence in homozygosity were viable and displayed no visible abnormalities, verifying the selected genome engineering approach (for wings, see Fig. 4F, ‘control’). As expected from the broad pattern of expression, flies devoid of the Mad-PA isoform were not viable. In contrast, flies able to express Mad-PA but not Mad-PB were viable and fertile. This is in agreement with previous findings demonstrating that uniformly expressed constructs comprising Mad-PA sequences fully restore the viability of *mad* mutants (Sekelsky et al., 1995). However, close examination of adult wings of Mad-PA-only flies revealed a substantially smaller size than that of control wings, suggesting that Mad-PB contributes to final organ size (Fig. 4F). Taken together, our analyses suggest a regulatory loop in which BMP signaling, starting at late larval wing development, activates the expression of a distinct Mad isoform, which contributes to proper organ development.

### Membrane tethering, but not GPI tethering, is essential for Dally function

**Fig. 5.**
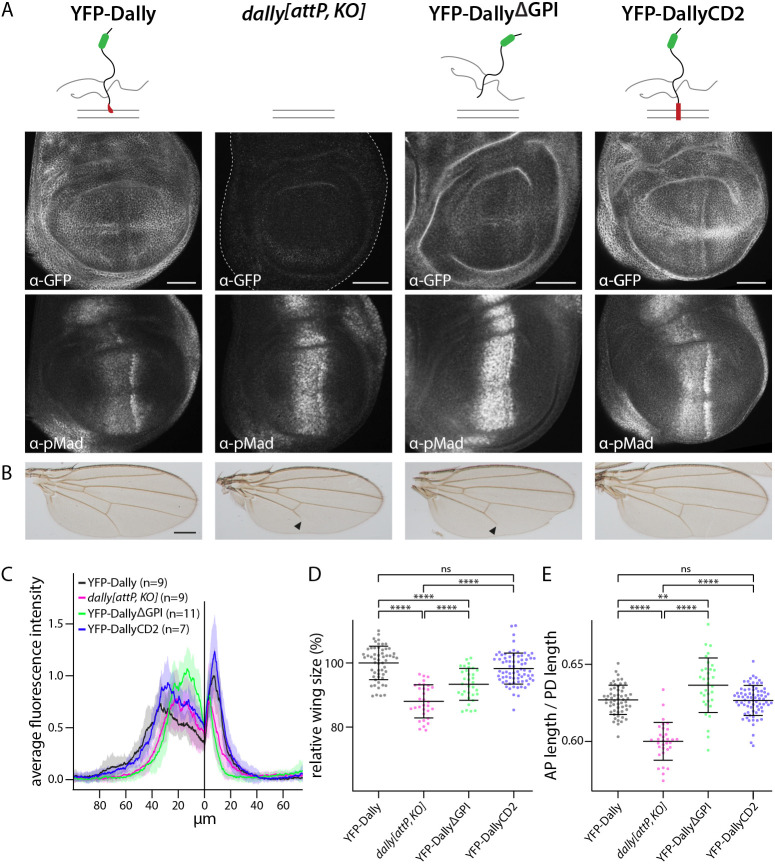
***In locus* structure-function analysis of Dally.** (A) Schematic illustration of the analyzed versions of Dally: YFP-Dally, *dally^[attp,KO]^*, YFP-Dally^ΔGPI^ and YFP-DallyCD2. Anti-GFP and anti-pMad staining of wing discs of larvae carrying the modified Dally alleles in homozygosity. Scale bars: 50 μm. (B) Adult wings of the Dally versions shown in A. All flies carried the respective modified Dally allele over the *dally^MH32^* null allele. Wings were collected from male flies. Black arrowheads indicate truncation of longitudinal vein 5. Scale bar: 250 μm. (C) Average pMad fluorescence intensity of wing discs homozygous for the Dally versions indicated in A, shown as mean±s.d. (D,E) Relative wing size (as a percentage) (D) and anterior-posterior (AP) length/proximal-distal (PD) length (E) of the indicated genotypes, shown as mean±s.d. Dots represent the measured values of individual wings isolated from male flies (YFP-Dally, *n*=56; *dally^[attp,KO]^*, *n*=32; YFP-Dally^ΔGPI^, *n*=33; YFP-DallyCD2, *n*=76). All flies carried the respective modified Dally allele over the *dally^MH32^* null allele. Statistical significance was analyzed by a two-tailed unpaired *t*-test with Welch's correction assuming unequal variances. ns, not significant, *P*>0.05; **P≤0.01; ****P≤0.0001.

We have recently used our engineering platforms to demonstrate that Dally, but not Dlp, is involved in the formation of the BMP signaling gradient (Simon et al., 2024). In addition, and in agreement with a recent study, we established that heparan sulfate modification is required for the function of Dally in gradient formation (Nakato et al., 2024). Here we extend these findings to interrogate another key structural feature of the protein: its membrane anchoring (Lin, 2004). Dally is tethered to the plasma membrane through a GPI anchor; however, the exact function of membrane anchoring or the specific requirement of the lipid anchoring has not been addressed under conditions of physiological expression. We used our *dally^[attP,KO]^* platform to address these questions. Consistent with previous reports (Franch-Marro et al., 2005; Lin and Perrimon, 1999; Nakato et al., 1995), *dally* mutant (*dally^[attP,KO]^*) flies displayed a number of defects, including very low hatching rates, absence of male external genitalia and characteristic defects in wing development. Adult wings were smaller, with a ‘pointed’ appearance (due to reduction of size along the anterior-posterior axis), and displayed distal truncations of longitudinal vein 5 (L5) (Fig. 5B,D,E; Fig. S1C). Reintegration of YFP-Dally resulted in flies that were fully viable and fertile (Simon et al., 2024). Wing discs of such flies displayed normal pMad distribution (Fig. 5A,C) and developed into adult wings of normal size, proportions and patterns (Fig. 5B,D,E; Fig. S2). Compared to YFP-Dally, reintegration of Dally with a C-terminal truncation that removes the GPI modification sites (YFP-Dally^ΔGPI^) resulted in a diffuse distribution in the disc epithelium, consistent with the protein being secreted and not confined to plasma membranes (Fig. 5A). Similar to *dally^[attP,KO]^* flies, YFP-Dally^ΔGPI^-expressing flies developed slowly and were semi-lethal, with only few larvae developing into adult, sterile flies. In addition, the secreted form of Dally failed to restore pMad distribution, L5 formation and wing size of *dally* mutants (Fig. 5). In contrast, reestablishment of membrane anchoring by fusing a CD2 transmembrane domain to the C-terminus of Dally (YFP-DallyCD2) reversed *dally* phenotypes. YFP-DallyCD2 flies were fully viable and fertile, and displayed normal pMad distribution and adult wing morphology (Fig. 5). Cumulatively, the findings suggest that anchoring, but not necessarily GPI anchoring, of Dally at the plasma membrane is important for Dally function.

### A complementary toolset for the manipulation of HA-tagged proteins

**Fig. 6.**
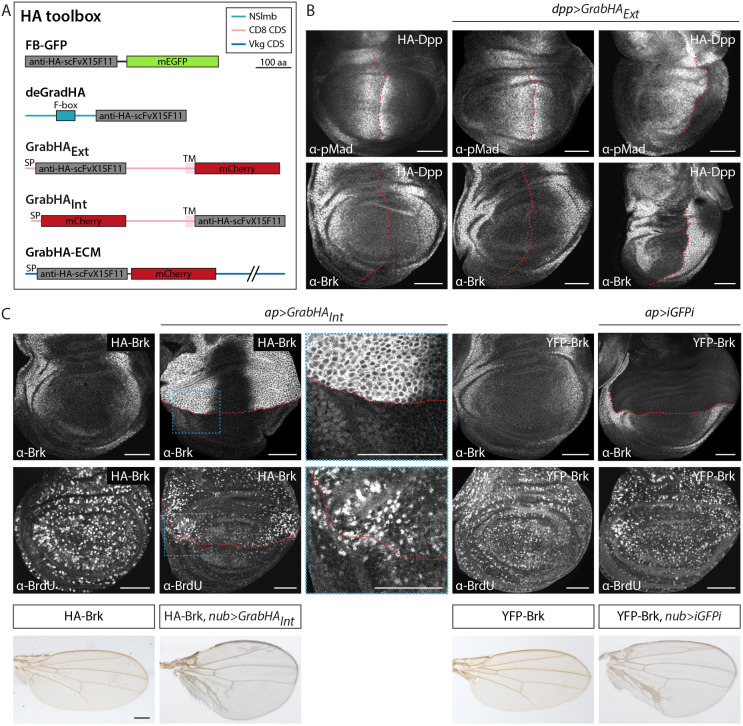
**The HA toolbox.** (A) Overview of the HA tools comprising FB-GFP, deGradHA, GrabHA_Ext_, GrabHA_Int_ and GrabHA-ECM. aa, amino acids; CDS, coding sequence; NSlmb, N-terminal part of Slmb; SP, signal peptide; TM, transmembrane domain. (B) Anti-pMad and anti-Brk staining of wing discs expressing HA-Dpp, GrabHA_Ext_ in *dpp* producing cells or a combination of both. The anterior-posterior compartment boundary is marked by red dashed lines. Scale bars: 50 μm. (C) Anti-Brk and anti-BrdU staining of wing discs expressing HA- or YFP-tagged versions of Brk either alone or in combination with the indicated tool in the dorsal compartment using ap-Gal4. Red dashed lines mark the dorsoventral compartment boundary. Adult wings of males carrying HA- or YFP-tagged versions of Brk either alone or in combination with overexpression of GrabHA_Int_ or iGFPi throughout the wing pouch using nub-Gal4 are shown below. Scale bars: 50 μm (wing discs); 250 μm (adult wings). Images are representative of at least five wing discs or 15 adult wings.

Recent studies have established a number of protein binder-based reagents that can be used to visualize and manipulate the activity of proteins that carry small peptide tags, including the HA epitope. Capitalizing on our collection of HA-tagged BMP components and inspired by a tool collection based on nanobodies against GFP and related fluorescent proteins (Caussinus et al., 2012; Harmansa et al., 2017; Matsuda et al., 2022), we generated and tested transgenic tools that are based on a single-chain antibody specifically recognizing the HA peptide called Frankenbody (anti-HA-scFvX15F11 or FB) (Zhao et al., 2019). In addition to FB-GFP and deGradHA, which we have shown recently to be useful for the visualization and degradation of proteins carrying single copies of the HA tag (Vigano et al., 2021), we generated constructs that localize FB to the outer (GrabHA_Ext_) or inner (GrabHA_Int_) surface of the plasma membrane, or at the basal lamina (GrabHA-ECM) (Fig. 6A). Such functionalized FB fusions can be useful for trapping or enriching intracellular or extracellular proteins to the corresponding compartments. Indeed, GrabHA_Ext_ expressed at the Dpp source efficiently trapped endogenously tagged HA-Dpp as shown by the compaction of the pMad gradient and the concomitant expansion of Brk expression (Fig. 6B). The effects were most prominent in the posterior compartment, where pMad was essentially absent and Brk was ectopically expressed in the whole compartment. The results, including the compartment asymmetry, are in line with a previous study using a different HA trap version (Matsuda et al., 2021). We also found that our GrabHA_Int_ efficiently localized nuclear HA-tagged proteins to the plasma membrane while simultaneously depleting them from the nucleus. Expression of GrabHA_Int_ in the dorsal compartment of the wing disc resulted in nuclear depletion and strong accumulation of HA-Brk to the plasma membrane (Fig. 6C). Consistent with the role of Brk in reducing proliferation in the wing disc (Restrepo et al., 2014; Schwank et al., 2008), depletion of Brk from the nucleus resulted in an increase of proliferation rates as judged by bromodeoxyuridine (BrdU) staining. Specifically, although proliferation rates were uniform throughout the wing imaginal disc and symmetric across compartments, trapping Brk at the plasma membrane resulted in a drastic increase in lateral proliferation in the manipulated, dorsal compartment (Fig. 6C). Furthermore, expression of GrabHA_Int_ throughout the wing pouch induced a strong and characteristic adult wing overgrowth (Barrio and Milán, 2017). The effects on proliferation rates and adult wing morphology were comparable to the effects of iGFPi-mediated depletion of YFP-Brk (Neumüller et al., 2012) (Fig. 6C). In addition to the manipulation of HA-tagged proteins with the HA-based toolset, YFP or FGT fusion proteins of our library were also susceptible to the previously established GFP nanobody-based tools (Caussinus et al., 2012; Harmansa et al., 2017); dorsal expression of deGradFP and morphotrap_Int_ resulted in efficient degradation and membrane translocation, respectively, of both nuclear Shn-FGT and cytosolic YFP-Smad2 (Fig. S7). Taken together, these results demonstrate that our library of modified TGF-β/BMP components is compatible with previously established and newly generated functionalized protein binder tools.

## DISCUSSION

Here, we present a comprehensive resource of genome-engineered components of the core *Drosophila* TGF-β/BMP signaling pathway. Our experimental strategy provides two distinct libraries that we expect to be useful in the field. The first collection comprises attP-insertions in 14 genes of the signaling pathway, which can not only be used as molecularly defined null alleles, but also serve as a ready-to-use genome-engineered platform for simple and efficient generation of variants of interest via ФC31-mediated integration. Our second collection capitalizes on this feature and consists of a number of functional epitope- or fluorescent-tagged components, which are expressed from the corresponding endogenous genomic loci. We demonstrate that the generated tagged proteins can be used for capturing tissue and subcellular distribution in selected tissues. Our analysis of the tagged proteins not only confirms the expected expression of the cognate genes, but also highlights some new aspects. For example, the levels of the receptors of the pathway are modulated along the anterior-posterior axis of the larval wing, a feature that has been previously established only for Tkv. Although we do not know whether this is a result of transcriptional or post-transcriptional regulation, these findings suggest that patterned expression of multiple receptors contributes in shaping the BMP activity gradient in this tissue. Our analyses also highlight variegated and dynamic expression of most components of the pathway in eggshell patterning and in the GSC niche.

Compared to recent efforts and advances in high-throughput epitope tagging, our gene-tailored approach, although not easily scalable, offers some advantages. Fosmid-based resources express epitope-tagged proteins from an additional gene copy that includes genomic regulatory regions (Sarov et al., 2016). Although extremely useful, these lines tend to be restricted to genes of small genomic size and might result in abnormal expression patterns due to omission of regulatory elements. Indicatively, tagged versions of genes with large and complex genomic loci, such as *tkv*, *dally*, *dlp* or *pent* are not represented in current libraries. In addition, the current fosmid project only used C-terminal tagging, which might affect protein function in some cases. Furthermore, the additional presence of the endogenous, untagged gene is incompatible with certain experiments and would require a simultaneous genetic removal of the untagged allele. At the same time, gene tagging using protein-trapping technologies is based on the insertion of artificial exon cassettes, thus excluding single-exon genes and drastically restricting the position of the tag within the protein sequence (Li-Kroeger et al., 2018; Nagarkar-Jaiswal et al., 2015; Venken et al., 2011). Our approach circumvents such problems and allows flexibility to specify the position of the tag for each gene individually. More importantly, the replacement of all or extensive sequence stretches of the coding regions by the attP cassette allows for efficient reintegration of gene variants, for example, for structure-function analyses. In the present study, we used this feature to address isoform and structural requirements of Mad and Dally, respectively.

Our studies addressing isoform utilization of *Mad* established patterned expression of a previously uncharacterized isoform, Mad-PB, during wing development. Interestingly, the expression of Mad-PB is under positive control of BMP signaling, which activates an alternative promoter of the gene at late stages of larval development. In contrast to the essential Mad-PA isoform, flies lacking Mad-PB had no gross abnormalities or developmental delays. However, such flies had significantly smaller wings, indicating that BMP-dependent activation of Mad-PB might be yet another regulatory loop in the system. At present, we can only speculate on the exact role of Mad-PB and its potential interaction with pMad. It is possible that BMP-dependent activation of Mad-PB boosts Mad levels during a crucial late larval/early pupal stage; however, there is no evidence that *Mad* expression levels are limited during wing development. In addition, the design of our Mad-PA-only construct abolishes Mad-PB production at the protein level but still allows transcriptional activation of the corresponding transcript; as this isoform comprises Mad-PA, a general elevation of Mad levels should still be possible in these flies. Alternatively, Mad-PB, with its N-terminal extension, might regulate wing growth through the regulation of pMad. It is conceivable that in this scenario, Mad-PB, either directly or after phosphorylation through the activated receptors, regulates the activity of pMad in gene regulation, diverts pMad activity towards different targets, or even blunts pMad activity towards signal termination.

We also used our genomic platforms to address structural features of Dally, a glypican that regulates the BMP signaling gradient in the wing disc by mechanisms that are not yet fully understood. Dally, like all glypicans, is anchored to the plasma membrane by a GPI anchor; however, the functional relevance of GPI anchoring has not been directly addressed. GPI anchors are important for protein compartmentalization at membrane domains and are involved in the regulation of endocytosis and protein trafficking (Mayor and Riezman, 2004; Sezgin et al., 2017). These processes have already been implicated in BMP signaling activation and ligand dispersion in the wing disc and elsewhere (Akiyama et al., 2008; Belenkaya et al., 2004; Cai et al., 2019 preprint; Morawa et al., 2015; Norman et al., 2016; Romanova-Michailidi et al., 2021; Simon et al., 2024). In addition, a recent synthetic biology approach suggested that Dally is directly involved in the transport of BMPs, through repeating rounds of GPI-mediated detachment of BMP-loaded Dally and reinsertion into the membrane of neighboring cells (Stapornwongkul and Vincent, 2021; Stapornwongkul et al., 2020). Our results clearly demonstrate that membrane anchoring of Dally is indeed important for its function, as expression of a protein with a C-terminal truncation removing the GPI modification sequences cannot rescue *dally* mutant phenotypes. However, restoring membrane tethering of the same construct by adding the transmembrane domain of the unrelated protein CD2 fully restores viability, fertility, and wing patterning and growth. Thus, based on these findings, membrane tethering but not GPI anchoring of Dally appears to be important for its function. Interestingly, it has also been suggested that Dlp, the second *Drosophila* glypican, does not require GPI anchoring for the regulation of Wg signaling based on overexpression studies (Yan et al., 2009).

Finally, and capitalizing on our collection of epitope- and fluorescent-tagged proteins, we tested whether our YFP/GFP- and HA-tagged proteins can be manipulated by recently established and newly generated functionalized protein binder tools. We demonstrate that YFP-tagged proteins, similar to GFP-tagged proteins, can indeed be efficiently recognized by the deGradFP and morphotrap_Int_ tools, resulting in degradation or trapping of the target protein, respectively (Caussinus et al., 2012; Harmansa and Affolter, 2018; Harmansa et al., 2017). Furthermore, our HA-tagged BMP components can also be destabilized by our recently established deGradHA tool, an anti-HA single chain antibody (FB; Zhao et al., 2019) fused to the F-box domain of Slmb, which channels proteins to ubiquitin/proteasomal degradation (Vigano et al., 2021). In addition, we established GrabHA_Ext_ and GrabHA_Int_ constructs, in which FB is tethered to the plasma membrane facing either the outside (GrabHA_Ext_) or the cytosol (GrabHA_Int_), and demonstrated that they can indeed efficiently trap and localize extracellular proteins (HA-Dpp) or even misroute and stabilize transcription factors at the plasma membrane (HA-Brk). We furthermore assume such tools to be functional when targeted to additional cellular domains and compartments.

In summary, we expect our collection of attP alleles as well as the library of epitope- and fluorescent-tagged TGF-β/BMP components, which allow expression at physiological levels and endogenous expression patterns, to greatly assist research in BMP and Activin signaling in different *Drosophila* tissues and during various processes. In addition, our newly established functionalized HA protein binder tools complement recent, similar collections and will certainly be useful for studies beyond BMP signaling (Harmansa et al., 2017; Kim et al., 2022; Xu et al., 2022).

## MATERIALS AND METHODS

### *Drosophila* lines

Df(2L)Exel6011 (7497), Df(2R)Exel6054 (7536), Df(2R)BSC270 (23166), Df(3R)ED5644 (9090), Df(3L)Exel6099 (7578), Df(3L)ED4414 (8702), Df(3L)ED4543 (8073), Df(2L)Exel7015 (7785), Df(3R)ED6361 (24143), Df(2R)Exel6060 (7542), Df(2R)X1 (1702), Df(3R)exel6176 (7655), Df(3R)BSC792 (27364), Dp(1;3)DC186 (30314), Dp(1;3)DC172 (30302), UAS-MadRNAi (31315), UAS-MedRNAi (31928), UAS-TkvRNAi (40937), UAS-GFPRNAi (iGFPi) (41556), ap-Gal4 (41245), Cre recombinase (851, 1092) and nos-Cas9 (54591, 78781, 78782) flies were provided by the Bloomington *Drosophila* Stock Center, and UAS-DadRNAi (42840), UAS-DallyRNAi (14136), UAS-DlpRNAi (10299), UAS-PutRNAi (37279), UAS-SaxRNAi (42457) and UAS-BaboRNAi (3825) by the Vienna *Drosophila* Resource Center. Other lines used were: vasa-ФC31 (Bischof et al., 2007); *dally^MH3^*^2^ (Franch-Marro et al., 2005); *pent^2^* (Vuilleumier et al., 2010); UAS_NSlmb-vhh-GFP4 (deGradFP) (Caussinus et al., 2012); UASTLOT_mCherry::CD8::vhh-GFP4 (morphotrap_Int_) (Harmansa et al., 2015); ap-Gal4, dpp-Gal4, hh-Gal4, ci-Gal4 (Konrad Basler, Institute of Molecular Biology, University of Zurich, Switzerland); nub-Gal4 (Walter Gehring, Department of Cell Biology, Biozentrum, University of Basel, Switzerland); and HA-Dpp (Bosch et al., 2017).

### Genome engineering and reintegration of tagged and/or modified gene versions

To replace specific parts of the genes encoding the TGF-β/BMP components with attP cassettes, genome engineering based on ends-out homologous recombination or CRISPR-induced homology-directed repair (HDR) was used. To manipulate *dally*, *pent*, *tkv* or *put*, homology arms flanking the targeted region of the respective gene were inserted into the pTV^Cherry^ targeting vector (*Drosophila* Genomics Resource Center, 1338) and the constructs were further integrated, mobilized as well as linearized as described previously (Baena-Lopez et al., 2013). Modified progeny with successful homologous recombination-mediated integration of the attP cassette was identified by the red eye color of the mini-white-containing selection cassette, which was subsequently removed using Cre recombination. For all other components, CRISPR-based HDR was applied. Guide RNA target sites flanking the region of interest were selected using the publicly available tools FlyCRISPR Optimal Target Finder (Gratz et al., 2014) and DRSC Find CRISPRs (Harvard Medical School; https://www.flyrnai.org/crispr/), and the genomic sequence at the target site was validated by sequencing. PCR-amplified homology arms were cloned into the pHD-dsRed-attP reintegration vector (Addgene, #51019; Gratz et al., 2014) and guide RNAs were cloned into the plasmid pCFD3-dU6:3 (for Dlp, Mad and Med) or pCFD4-U6:1_U6:3 (for Babo, Brk, Dad, Sax, Shn, Smad2 and Wit) (Addgene, #49410 and #49411; Port et al., 2014). Co-injection of the homology arms and guide RNA-containing plasmids into *nos-Cas9*-expressing flies resulted in HDR, and successful integration of the attP cassette was identified by expression of the 3×P3-dsRed marker, which was then excised using Cre recombination. Primers used for amplification of the homology arms as well as the generation of the guide RNA plasmids are listed in Table S1, and deletion strategies for all components are schematically depicted in Fig. 1B and Fig. S1A. Introduced deletions were verified by sequencing (Fig. S1B).

In the second step, standard ФC31/attB integration was used to reintegrate either tagged and/or modified gene versions (e.g. for Mad and Dally) into the attP sites using the RIVwhite vector (*Drosophila* Genomics Resource Center, 1330; Baena-Lopez et al., 2013). Depending on gene architecture and the introduced deletion, generated reintegration vectors contained either the deleted sequence or full-length cDNA. Primers used for the generation of the reintegration vectors are listed in Table S2, and schematic depictions of the reintegration strategies can be found in Fig. 1B and Fig. S1A. All generated constructs were verified by sequencing.

Some of the generated attP,KO lines as well as some reintegration lines have been previously published: *tkv^[attP, KO]^*, Tkv-3xHA, *pent^[attP, KO]^* and YFP-Pent (Norman et al., 2016; Tracy Cai et al., 2019); Tkv-HAeGFP (Vigano et al., 2021); *brk^[attP, KO]^*, HA-Brk, *shn^[attP, KO]^* and Shn-HA (Vuilleumier et al., 2022); and dally*^[attP, KO]^*, YFP-Dally, dlp*^[attP, KO]^* and HA-Dlp (Simon et al., 2024).

### Generation of the HA toolbox

pUASTLOTattB_anti-HA_fb_GFP (referred to as FB-GFP) and pUASTLOTattB_deGradHA (referred to as deGradHA) have been previously described (Vigano et al., 2021). pUASTLOTattB_GrabHA_Ext_ and pUASTLOTattB-GrabHA_Int_ were generated by replacing vhh-GFP4 of pUASTLOTattB_vhh-GFP4::CD8::mCherry (Addgene #163917; Harmansa et al., 2015) or pUASTLOTattB_mCherry::CD8::vhh-GFP4 (Addgene #163930; Harmansa et al., 2017), respectively, with the PCR-amplified Frankenbody anti-HA-scFvX15F11_mEGFP (referred to as anti-HA_fb) (Vigano et al., 2021; Zhao et al., 2019). For pUASTLOTattB_GrabHA-ECM, vhh-GFP4 was cut out of pUASTLOTattB_vhh-GFP4::Vkg::mCherry (Addgene #163929; Harmansa et al., 2017) and anti-HA_fb was integrated. All generated constructs were verified by sequencing. Flies carrying UASTLOT_anti-HA_fb_GFP, UASTLOT_deGradHA, UASTLOT_GrabHA_Ext_, UASTLOT_GrabHA_Int_ or UASTLOT_GrabHA-ECM on chromosome 3L at position 68A4 (attP2) were generated by standard ФC31/attB transgenesis. Primers used for the HA toolbox plasmids are listed in Table S3.

### Dissection and antibody staining of *Drosophila* tissues

For larval wing imaginal disc immunostaining, third instar larvae were collected in phosphate-buffered saline (PBS) and cut into half. The posterior end was discarded and the remaining anterior part was inverted. The carcasses were roughly cleaned from excessive tissue and fixed in 4% paraformaldehyde (PFA) for 20 min. Carcasses were rinsed twice in 0.1% Triton X-100 in PBS (PBSTx), washed two times for 10 min in PBSTx and incubated for 1 h in blocking solution (5% normal goat serum in PBSTx). Samples were then incubated with primary antibodies in blocking solution overnight at 4°C. On the next day, carcasses were rinsed twice, washed three times for 20 min in PBSTx and incubated with fluorescently labelled secondary antibodies and Hoechst 33342 in blocking solution for at least 2 h. Samples were rinsed and washed three times for 10 min in PBSTx. After rinsing with PBS, the wing discs were fine-dissected and mounted in VECTASHIELD Antifade Mounting Medium (Biozol).

For dissection of wing discs from staged larvae, adults were kept in cages with grape juice plates with yeast paste to lay eggs for up to 2 h at 25°C. At about 22-24 h after egg laying, hatched first instar larvae were transferred to new plates (up to 25 per plate) and maintained at 25°C until the desired age. Wing discs were then isolated, transferred to 4% PFA for 20 min and immunostained as described above.

For prepupal wings, white prepupae (0 h after puparium formation) were collected and incubated at 25°C until the desired age. Wings were isolated from the prepupae, transferred to 4% PFA, fixed for 20 min and immunostained as the larval wing discs.

Ovaries were isolated from 2- to 4-day-old female flies in PBS and were carefully opened to separate individual ovarioles, which were then fixed and stained as described for the wing discs.

BrdU labeling of larval wing imaginal discs was performed as follows: third instar larvae were dissected as described above, transferred into S2 medium (Gibco℣) and BrdU (0.1 mg/ml) was added to the medium. After 15 min incubation on a rocking platform, carcasses were fixed in 4% PFA for 20 min, rinsed three times in PBSTx and incubated 45 min in 2 N hydrochloric acid. After two short incubations with Na_3_BO_3_ (0.1 M, pH 8.5) for 2 min, samples were rinsed and washed twice in PBSTx for 10 min. A 20-min incubation in blocking solution was followed by incubation with an anti-BrdU antibody overnight at 4°C. On the next day, samples were rinsed twice, washed three times for 10 min with PBSTx and blocked for 20 min in blocking solution. Fluorescently labelled secondary antibodies and Hoechst 33342 in blocking solution were added for at least 2 h. Further steps after secondary antibody incubation were performed as described above.

The following antibodies and reagents were used in this study: chicken anti-GFP (1:1000, Abcam, ab13970), rabbit anti-GFP (1:400, Thermo Fisher Scientific, G10362), rat anti-HA (1:200, Roche, 11867423001), mouse anti-Dlg [1:50, Developmental Studies Hybridoma Bank (DSHB), AB_528203], rabbit anti-pMad (1:500, Abcam, ab52903), rabbit anti-pMad (1:500, Cell Signaling Technology, 9516), guinea pig anti-Brk (1:500, a gift from Hilary Ashe, Faculty of Life Sciences, University of Manchester, UK), mouse anti-Wg (1:40, DSHB, AB_528512), mouse anti-Ptc (1:40, DSHB, AB_528441), rat anti-DCad (1:50, DSHB, AB_528120), mouse anti-Hts (1:10, DSHB, AB_528070), mouse anti-BrdU antibody (1:100, BD Biosciences, 347580), Alexa Fluor-conjugated secondary antibodies (1:500, Thermo Fisher Scientific, A-11006, A-11029, A-11031, A-11034, A-11036, A-11039, A-11073, A-11077, A-21052, A-21094) and Hoechst 33342 (1:5000, Invitrogen).

### Imaging and image processing

All immunostained samples were imaged with a ZEISS LSM 880 laser scanning confocal microscope (Life Imaging Center, Hilde Mangold Haus, University of Freiburg), which provided the additional opportunity to use a Airyscan detector or the FAST Airyscan mode for image acquisition. Whenever Airyscan detection was used, raw data were processed using the Airyscan processing function in the ZEISS ZEN Black 2.3 software. Images were analyzed and processed with Fiji and Adobe Photoshop. Figures were prepared using Adobe Illustrator.

### Quantification of pMad intensity

To obtain average intensity profiles of different samples of one genotype, average-intensity *z*-projections of three consecutive *z*-slices were generated using Fiji and signal intensity profiles along the anterior-posterior wing disc axis were obtained in the dorsal compartment using identically sized boxes and the plot profile function in Fiji. The measured values were transferred to Excel (Microsoft) and values of different samples of the same genotype were aligned along the anterior-posterior compartment boundary based on Ptc and/or pMad stainings, and an average intensity profile was generated using the script wing_disc-alignment.py (Simon et al., 2024). Average intensity curves of different genotypes were then aligned and compared using the script wingdisc_comparison.py (Simon et al., 2024), normalizing the data with the smallest value of each experimental condition (normalization option ‘*n*’). The figure of the average intensity profile plots containing the standard deviations for all compared genotypes was then prepared using Adobe Illustrator.

### Adult wing preparation, imaging and quantification

Adult flies were collected in isopropanol, one wing per fly was dissected and wings were mounted in Euparal mounting medium (Carl Roth) on a glass slide and covered with a coverslip. Images were acquired on a Leica MZ Apo stereomicroscope using a ZEISS Axiocam 305 color camera. The sex of the flies used for wing analysis is indicated in the respective figure legends.

Wing area and/or anterior-posterior and proximal-distal lengths were measured using Fiji or Adobe Illustrator and values were transferred to Excel. Plots were generated using Prism (GraphPad) and adapted in Adobe Illustrator. Statistical significance was analyzed by a two-tailed unpaired *t*-test with Welch's correction assuming unequal variances using Prism, with *P*>0.05 taken as not significant, *P≤0.05, **P≤0.01, ***P≤0.001 and ****P≤0.0001.

## Supplementary Material



10.1242/develop.204222_sup1 Supplementary information

Table S1. Primers to generate plasmids containing homology arms and guide RNAs

Table S2. Primers to generate reintegration vectors

Table S3. Primers to generate HA toolbox plasmids
