# Supplementary material for: A genome-engineered tool set for Drosophila TGF-β/BMP signaling studies
Source: Development. 2024 Nov 18;151(22):dev204222. doi: 10.1242/dev.204222 (PMC11607693; doi:10.1242/dev.204222)
Supplement: Supplementary information [file develop-151-204222-s1.pdf]

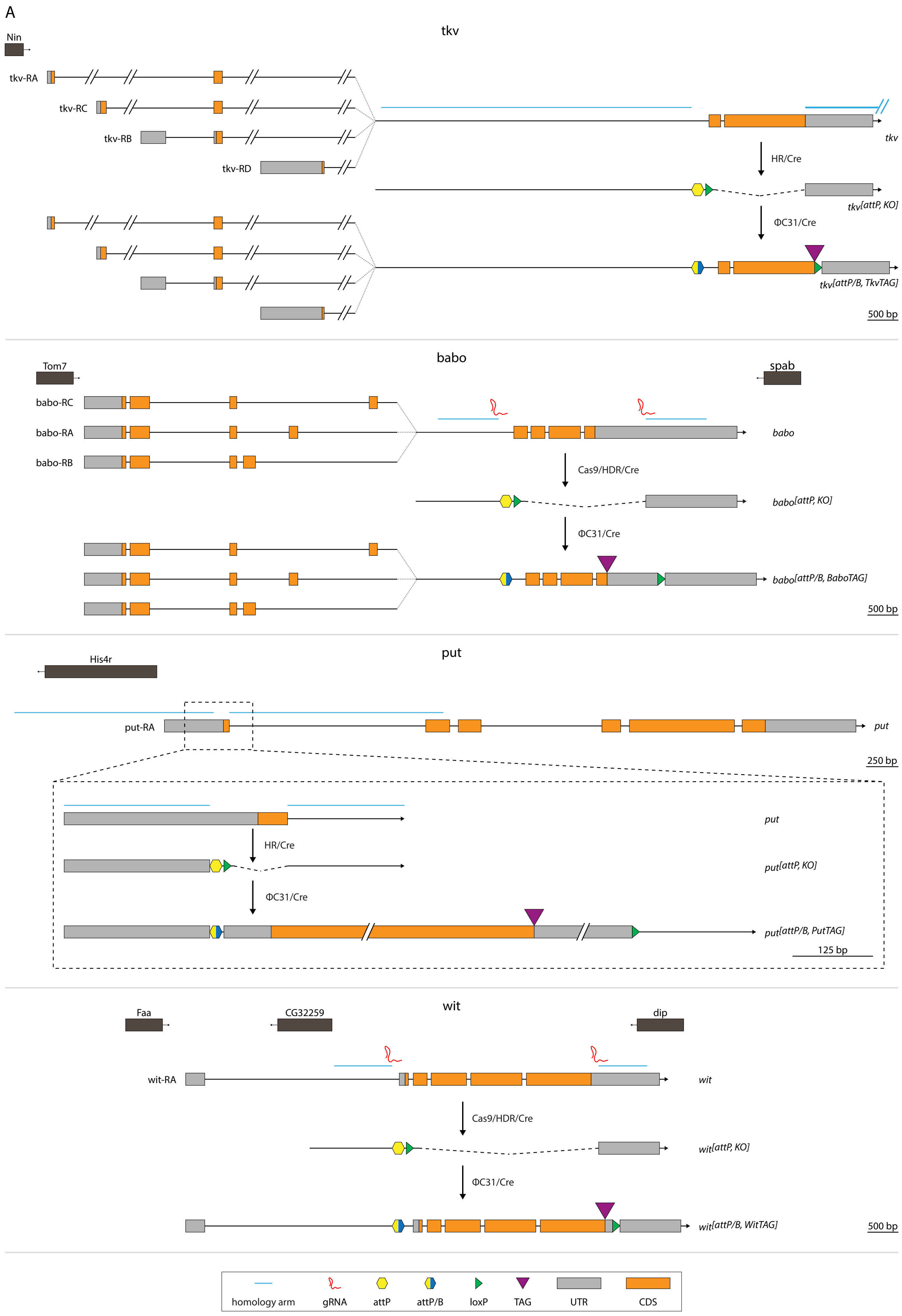


Development • Supplementary information

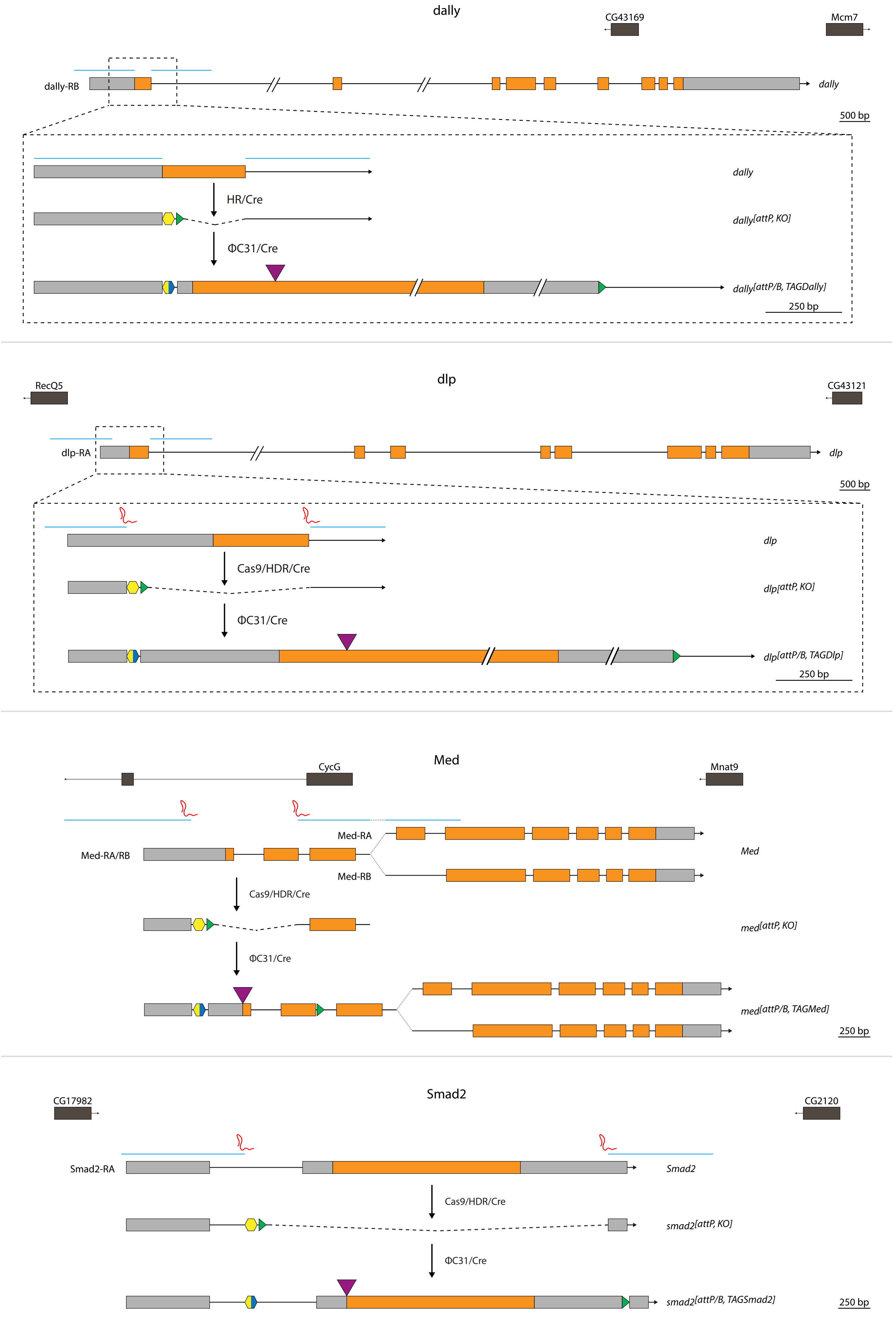


Development • Supplementary information

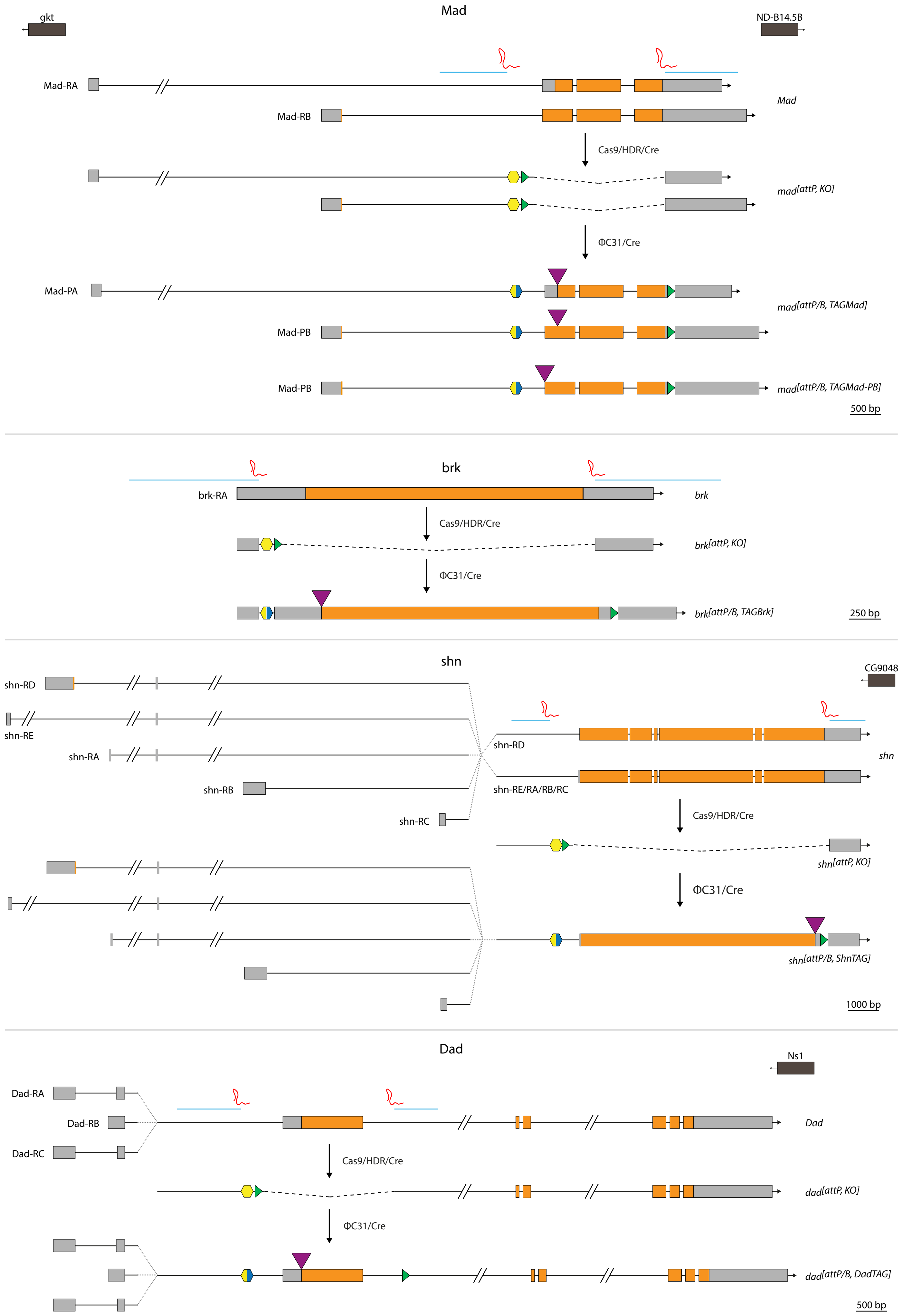


Development • Supplementary information

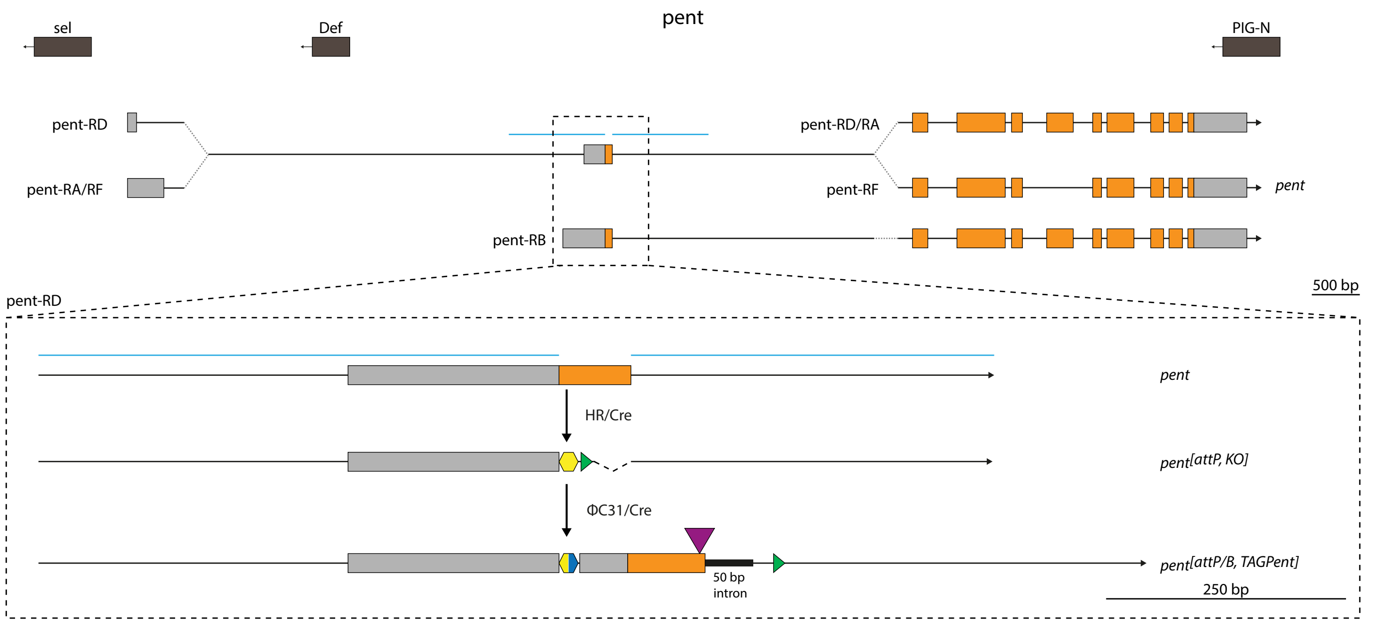


B

| | | | | | | | |
|---|---|---|---|---|---|---|---|
| *tkv[attP, KO]* | TGGTTTAAAGGTGGGGATAAGACCAACCCACTCGTCAAC | GGTACC | GTAGTGCCCCAACTGGGGTAACCTTTGAGTTCTCTCAGTTGGGGGCGTAG | TGTACC | ATAACTTCGTATAATGTATGCTATACGAAGTTAT | CACTAGTAAAGATCT | GCAGCTGTTTGTAGTC |
| *sax[attP, KO]* | ATTAAAAGCTGCCCG | GCGGCCGCGGACATATGCACACCTGCGATC | GTAGTGCCCCAACTGGGGTAACCTTTGAGTTCTCTCAGTTGGGGGCGTAG | | ATAACTTCGTATAATGTATGCTATACGAAGTTAT | AGAAGAGCACTAGT | GGTATATTATTCAAAGC |
| *babo[attP, KO]* | AGTATTATTAACCGC | GCGGCCGCGGACATATGCACACCTGCGATC | GTAGTGCCCCAACTGGGGTAACCTTTGAGTTCTCTCAGTTGGGGGCGTAG | | ATAACTTCGTATAATGTATGCTATACGAAGTTAT | AGAAGAGCACTAGA | GGTATCCATATTGCTGG |
| *put[attP, KO]* | TAATATTAACAGTACCCAGTTATTCCATTCCACTGCACC | GGTACC | GTAGTGCCCCAACTGGGGTAACCTTTGAGTTCTCTCAGTTGGGGGCGTAG | TGTACC | ATAACTTCGTATAATGTATGCTATACGAAGTTAT | CACTAGT | GTATTGCACTGCGAGTGTGTGGGT |
| *wit[attP, KO]* | TTTTTGTGGTGGGGA | GCGGCCGCGGACATATGCACACCTGCGATC | GTAGTGCCCCAACTGGGGTAACCTTTGAGTTCTCTCAGTTGGGGGCGTAG | | ATAACTTCGTATAATGTATGCTATACGAAGTTAT | AGAAGAGCA | GAAGACTTGAATCGCTTCCTGG |
| *dally[attP, KO]* | TGTGTCTGCACACAGAAATCACCCATCAAAA | CATATGCAGGTACC | GTAGTGCCCCAACTGGGGTAACCTTTGAGTTCTCTCAGTTGGGGGCGTAG | TGTACC | ATAACTTCGTATAATGTATGCTATACGAAGTTAT | CACTAGT | GTAAAGTTCACGCCATCCATCCGT |
| *dlp[attP, KO]* | ACACCCGACAACCATG | CGGCCGCGGACATATGCACACCTGCGATC | GTAGTGCCCCAACTGGGGTAACCTTTGAGTTCTCTCAGTTGGGGGCGTAG | | ATAACTTCGTATAATGTATGCTATACGAAGTTAT | AGAAGAGCACTAGA | TGTCCGATATTATATAC |
| *mad[attP, KO]* | CTCAAAAATACCCCC | GCGGCCGCGGACATATGCACACCTGCGATC | GTAGTGCCCCAACTGGGGTAACCTTTGAGTTCTCTCAGTTGGGGGCGTAG | | ATAACTTCGTATAATGTATGCTATACGAAGTTAT | AGAAGAGCACTAGA | CTCAATGGAGACGGAAG |
| *med[attP, KO]* | CCCCAAGCCCACT | CAGCGGCCGCGGACATATGCACACCTGCGATC | GTAGTGCCCCAACTGGGGTAACCTTTGAGTTCTCTCAGTTGGGGGCGTAG | | ATAACTTCGTATAATGTATGCTATACGAAGTTAT | AGAAGAGCACTAGT | AGTCTCTCAGACTTTCT |
| *smad2[attP, KO]* | TGCCCACAGATTAGG | GCGGCCGCGGACATATGCACACCTGCGATC | GTAGTGCCCCAACTGGGGTAACCTTTGAGTTCTCTCAGTTGGGGGCGTAG | | ATAACTTCGTATAATGTATGCTATACGAAGTTAT | AGAAGAGCACTAGA | ATGTGGGATATATATCT |
| *brk[attP, KO]* | ATCCGATCTATACCA | GCGGCCGCGGACATATGCACACCTGCGATC | GTAGTGCCCCAACTGGGGTAACCTTTGAGTTCTCTCAGTTGGGGGCGTAG | | ATAACTTCGTATAATGTATGCTATACGAAGTTAT | AGAAGAGCACTAGA | AATGGCAGTCACTGGGC |
| *shn[attP, KO]* | TTATAATCCCATTGC | GCGGCCGCGGACATATGCACACCTGCGATC | GTAGTGCCCCAACTGGGGTAACCTTTGAGTTCTCTCAGTTGGGGGCGTAG | | ATAACTTCGTATAATGTATGCTATACGAAGTTAT | AGAAGAGCA | CTAGAGCTAGCCTATAAGACCC |
| *pent[attP, KO]* | GTTAATTGACGAATTCGAACTGCTAAGTGAA | CATATGCAGGTACC | GTAGTGCCCCAACTGGGGTAACCTTTGAGTTCTCTCAGTTGGGGGCGTAG | TGTACC | ATAACTTCGTATAATGTATGCTATACGAAGTTAT | CACTAGT | GTAAGTTTACTTTCG |
| *dad[attP, KO]* | CAAGCGTATTTGGGC | GCGGCCGCGGACATATGCACACCTGCGATC | GTAGTGCCCCAACTGGGGTAACCTTTGAGTTCTCTCAGTTGGGGGCGTAG | | ATAACTTCGTATAATGTATGCTATACGAAGTTAT | AGAAGAGCACTAGA | ATGTATTCGGTGGCGCC |

genomic sequence  MCS remnants  attP site  loxP site  MCS remnants  genomic sequence

C

| | tested over | result |
|---|---|---|
| *tkv[attP, KO]* | Df(2L)Exel6011 | lethal |
| *sax[attP, KO]* | Df(2R)Exel6054 | lethal |
| *babo[attP, KO]* | Df(2R)BSC270 | lethal |
| *put[attP, KO]* | Df(3R)ED5644 | lethal |
| *wit[attP, KO]* | Df(3L)Exel6099 | lethal |
| *dally[attP, KO]* | Df(3L)ED4414, *dally[MH32]* | viable; phenotypic manifestation |
| *dlp[attP, KO]* | Df(3L)ED4543 | lethal |
| *mad[attP, KO]* | Df(2L)Exel7015 | lethal |
| *med[attP, KO]* | Df(3R)ED6361 | lethal |
| *smad2[attP, KO]* | - | hemizygous lethal; viability restored with Dp(1;3)DC186 |
| *brk[attP, KO]* | - | hemizygous lethal; viability restored with Dp(1;3)DC172 |
| *shn[attP, KO]* | Df(2R)Exel6060 | lethal |
| *pent[attP, KO]* | Df(2R)X1, *pent[2]* | viable; phenotypic manifestation |
| *dad[attP, KO]* | Df(3R)Exel6176, Df(3R)BSC792 | viable |

C'

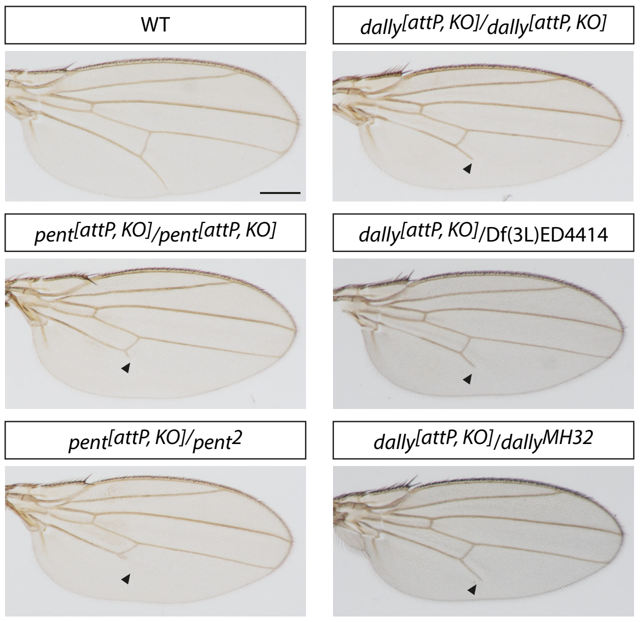


Development • Supplementary information

**Fig. S1. Genome engineering of components of the TGF-β/BMP signaling pathway.** (A) Simplified overview of two-step genome engineering strategies for all modified TGF-β/BMP components. Intermediate steps bevor removal of selection markers are not shown (compare Fig. 1B). Note that in case of Dally, Pent, Tkv and Put homologous recombination was performed as described in (Baena-Lopez et al., 2013) instead of CRISPR/Cas9 based homology-directed repair to generate the attP, KO lines. Color code as shown in box under *wit* strategy. HR = homologous recombination, HDR = homology-directed repair, UTR = untranslated region, CDS = coding sequence, bp = base pairs. (B) Exact sequences of modified loci after integration of the attP site and removal of the selection marker. Color code: blue = genomic sequence, yellow = attP site, green = loxP site, black = remnants of multiple cloning site (MCS). (C) Generated knockout alleles were genetically verified by testing over the indicated fly lines (chromosomal deficiencies, previously described null alleles or mutants, chromosomal duplications for genes on X chromosome). *dally*$^{[attP,\ KO]}$ and *pent*$^{[attP,\ KO]}$ are viable over the tested chromosomes and show wing phenotypes as shown in C'. Wings are smaller than wild-type (WT) wings and show truncations of longitudinal vein 5 (indicated by black arrowheads). Wings were isolated from female flies. Scale bar: 250 µm.

Development • Supplementary information

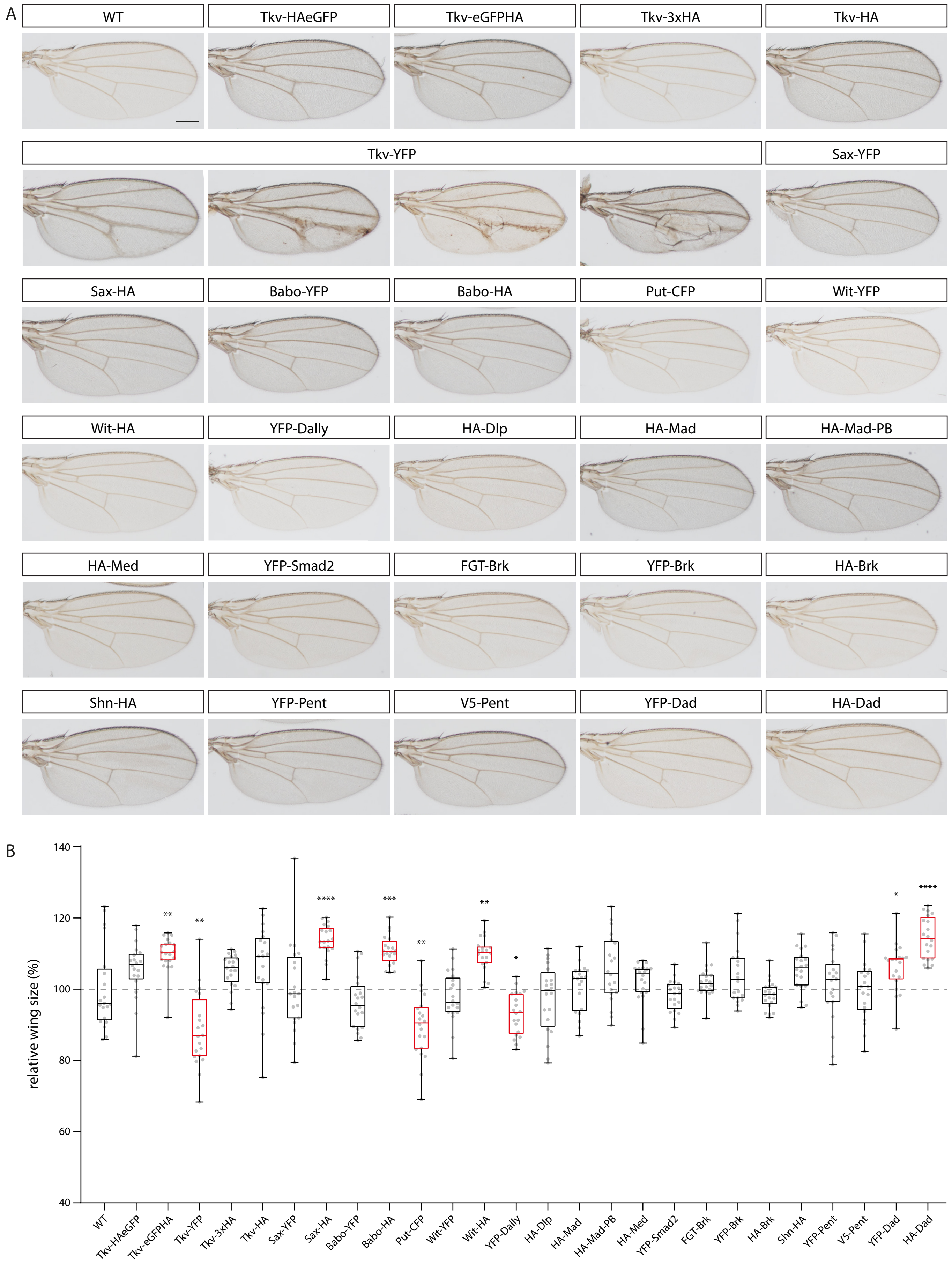


**Fig. S2. *In-locus* modified TGF-β/BMP components support wing development.** (A) Adult wings of male wild-type (WT) flies and male flies carrying the tagged components in homozygosity. All wings show normal morphology and patterning except for wings of flies homozygous for Tkv-YFP, which frequently show thickening of veins and blisters. Scale bar: 250 µm. (B) Graph shows distribution of relative wing size (%) as boxplot for the indicated components. Dots represent measured size of individual wings collected from male flies (n = 20 for all). Statistical significance was analyzed by a two-tailed unpaired t-test with Welch's correction assuming unequal variances comparing the wing size of the tagged components to WT. Instances deviating significantly from the control are highlighted in red. $p>0.05$ = ns (not labelled in the graph), $p \leq 0.05$ = *, $p \leq 0.01$ = **, $p \leq 0.001$ = *** and $p \leq 0.0001$ = ****.

Development • Supplementary information

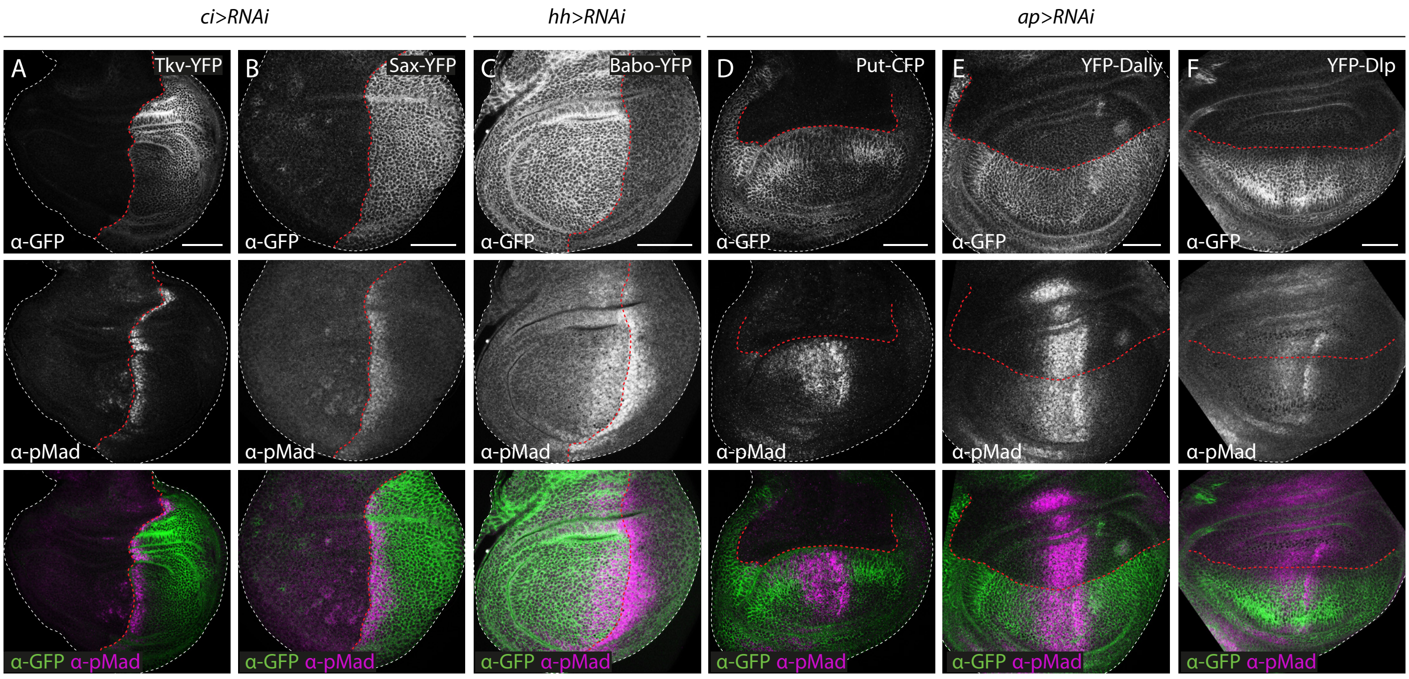


**Fig. S3. Compartment specific depletion of tagged TGF-β/BMP receptors and glypicans.** (A-F) Anti-GFP and anti-pMad stainings visualize the distribution of receptors and glypicans in the wing imaginal disc as well as the effect on pMad upon RNAi-mediated depletion of Tkv (A), Sax (B), Babo (C), Put (D), Dally (E) or Dlp (F). Gene-specific RNAi is active either in the anterior compartment using ci-Gal4 (A, B), in the posterior compartment using hh-Gal4 (C) or in the dorsal compartment using ap-Gal4 (D-F). Note that depletion of the BMP receptors Tkv, Sax and Put, but not the Activin receptor Babo, results in a reduction of pMad signal. In addition, dorsal depletion of Dally results in a constriction of the pMad extend in the same compartment. As shown recently, this is not the case for Dlp, which is not involved in the generation of the pMad gradient in the wing imaginal disc (Simon et al., 2024). Red dashed line indicates anterior-posterior (A-C) or dorso-ventral (D-F) compartment boundary. Scale bars: 50 µm.

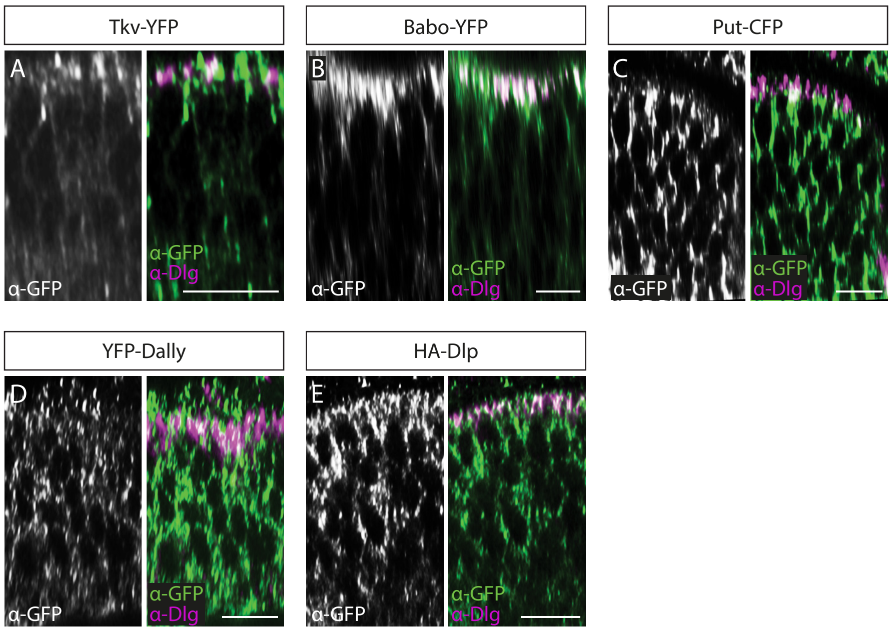


**Fig. S4. Subcellular localization of tagged receptors and glypicans**. (A-E) Distribution of YFP-, CFP- or HA-tagged components in the disc proper visualized by anti-GFP or anti-HA staining in relation to Discs large (Dlg) (magenta). Scale bar: 10 μm.

Development • Supplementary information

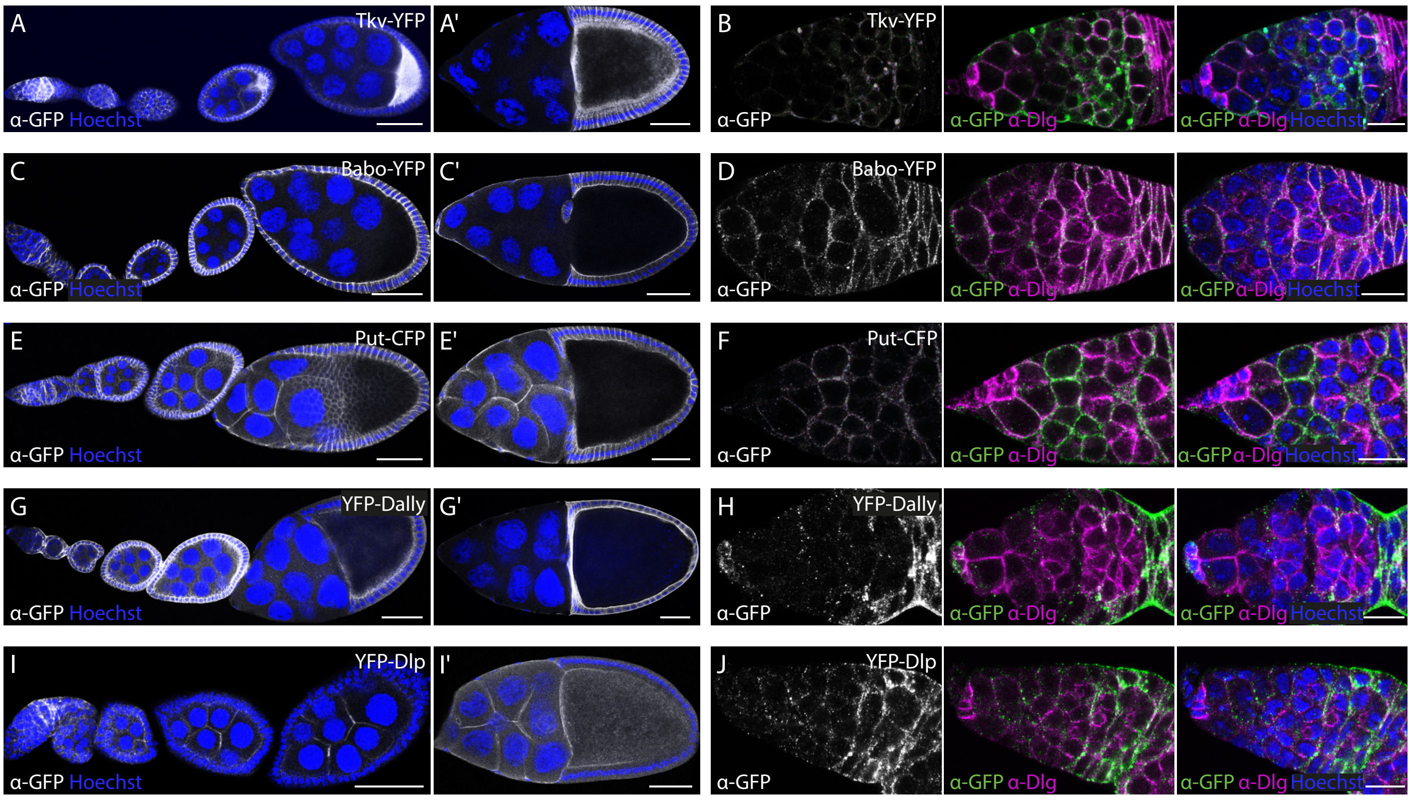


**Fig. S5. Distribution of tagged receptors and glypicans during oogenesis.** (A-J) Anti-GFP staining of YFP- or CFP-tagged components shows their distribution during oogenesis. Nuclei are visualized by Hoechst (blue) and Discs large (Dlg) is shown in magenta. Scale bars: 50 µm (A, C, E, G, I), 10 µm (B, D, F, H, J).

Development • Supplementary information

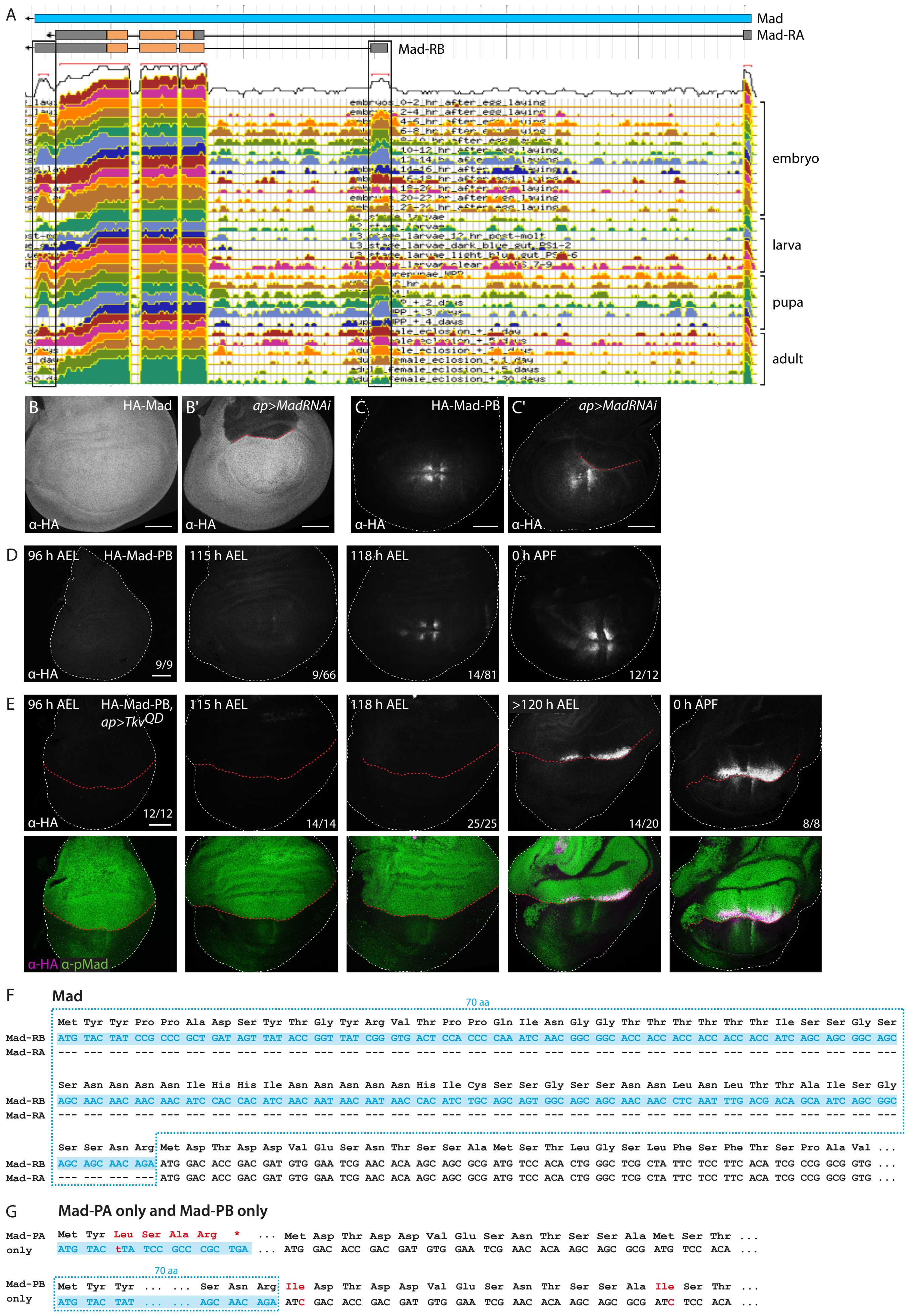
A
Mad
Mad-RA
Mad-RB
embryo
larva
pupa
adult
B HA-Mad
B' ap>MadRNAi
C HA-Mad-PB
C' ap>MadRNAi
α-HA
D 96 h AEL HA-Mad-PB
115 h AEL
118 h AEL
0 h APF
9/9
9/66
14/81
12/12
E 96 h AEL HA-Mad-PB, ap>TkvQD
115 h AEL
118 h AEL
>120 h AEL
0 h APF
12/12
14/14
25/25
14/20
8/8
α-HA α-pMad
F Mad
70 aa
Met Tyr Tyr Pro Pro Ala Asp Ser Tyr Thr Gly Tyr Arg Val Thr Pro Pro Gln Ile Asn Gly Gly Thr Thr Thr Thr Thr Thr Ile Ser Ser Gly Ser
Mad-RB ATG TAC TAT CCG CCC GCT GAT AGT TAT ACC GGT TAT CGG GTG ACT CCA CCC CAA ATC AAC GGC GGC ACC ACC ACC ACC ACC ACC ATC AGC AGC GGC AGC
Mad-RA --- --- --- --- --- --- --- --- --- --- --- --- --- --- --- --- --- --- --- --- --- --- --- --- --- --- --- --- --- --- --- --- ---
Ser Asn Asn Asn Asn Ile His His Ile Asn Asn Asn Asn Asn His Ile Cys Ser Ser Gly Ser Ser Asn Asn Leu Asn Leu Thr Thr Ala Ile Ser Gly
Mad-RB AGC AAC AAC AAC AAC ATC CAC CAC ATC AAC AAT AAC AAT AAC CAC ATC TGC AGC AGT GGC AGC AGC AAC AAC CTC AAT TTG ACG ACA GCA ATC AGC GGC
Mad-RA --- --- --- --- --- --- --- --- --- --- --- --- --- --- --- --- --- --- --- --- --- --- --- --- --- --- --- --- --- --- --- --- ---
Ser Ser Asn Arg Met Asp Thr Asp Asp Val Glu Ser Asn Thr Ser Ser Ala Met Ser Thr Leu Gly Ser Leu Phe Ser Phe Thr Ser Pro Ala Val ...
Mad-RB AGC AGC AAC AGA ATG GAC ACC GAC GAT GTG GAA TCG AAC ACA AGC AGC GCG ATG TCC ACA CTG GGC TCG CTA TTC TCC TTC ACA TCG CCG GCG GTG ...
Mad-RA --- --- --- --- ATG GAC ACC GAC GAT GTG GAA TCG AAC ACA AGC AGC GCG ATG TCC ACA CTG GGC TCG CTA TTC TCC TTC ACA TCG CCG GCG GTG ...
G Mad-PA only and Mad-PB only
Mad-PA only Met Tyr Leu Ser Ala Arg * ... Met Asp Thr Asp Asp Val Glu Ser Asn Thr Ser Ser Ala Met Ser Thr ...
ATG TAC tTA TCC GCC CGC TGA ... ATG GAC ACC GAC GAT GTG GAA TCG AAC ACA AGC AGC GCG ATG TCC ACA ...
70 aa
Mad-PB only Met Tyr Tyr ... ... Ser Asn Arg Ile Asp Thr Asp Asp Val Glu Ser Asn Thr Ser Ser Ala Ile Ser Thr ...
ATG TAC TAT ... ... AGC AAC AGA ATC GAC ACC GAC GAT GTG GAA TCG AAC ACA AGC AGC GCG ATC TCC ACA ...

**Fig. S6. *Mad* isoform analysis.** (A) RNA-Seq expression data for different developmental stages by the ModENCODE project predicts two transcript isoforms, Mad-RA and Mad-RB (Graveley et al., 2011). Screenshot from Flybase JBrowse and adapted. Black boxes highlight Mad-RB specific reads, labels at right indicate developmental stage. (B, C) Anti-HA staining of endogenously tagged Mad versions in late 3rd instar wing discs. (B', C') RNAi-mediated depletion of Mad in the dorsal wing disc compartment using ap-Gal4 verifies specificity of the observed staining. Red dashed line indicates dorso-ventral compartment boundary. Scale bars: 50 µm. (D) Anti-HA staining of HA-Mad-PB in staged wing discs. No signs of expression are visible at 96 h After Egg Laying (AEL), while faint signal starts to appear in a small fraction of discs (9 out of 66) at 115 h AEL. At 118 h AEL, robust staining in four patches is visible in a subset of discs (14 out of 88), while all wings are positive for Mad-PB at 0 h After Puparium Formation (APF). Scale bar: 50 µm. (E) Anti-HA and anti-pMad immunostaining in staged discs expressing $Tkv^{QD}$ in the dorsal compartment using ap-Gal4. Increasing BMP activity results in an expansion of the two dorsal patches of HA-Mad-PB only shortly before puparium formation (>120 h AEL) and at the onset of pupariation (0 h APF). The patches of HA-Mad-PB are not visible at 118 h AEL under these conditions, probably due the developmental delay caused by BMP overactivation as reported before (Setiawan et al., 2018). Red dashed line indicates dorso-ventral compartment boundary. Scale bar: 50 µm. (F) Start of DNA and amino acid sequences of the two Mad isoforms. Mad-PB is an in-frame N-terminal extension of Mad-PA, containing 70 amino acids (marked in blue) upstream of the Mad-RA start codon. aa = amino acids. (G) To generate flies which exclusively contain one of the isoforms, we reintroduced mutated versions of the genomic sequence of *Mad* into *mad*$^{[attP,\ KO]}$ flies. For Mad-PA only flies, we introduced a frameshift into Mad-RB by inserting a single nucleotide. For Mad-PB only flies, we mutated the Mad-RA start codon (along with a second nearby ATG) by exchanging them with ATC (isoleucine). Changes compared to wild-type sequence are highlighted in red.

Development • Supplementary information

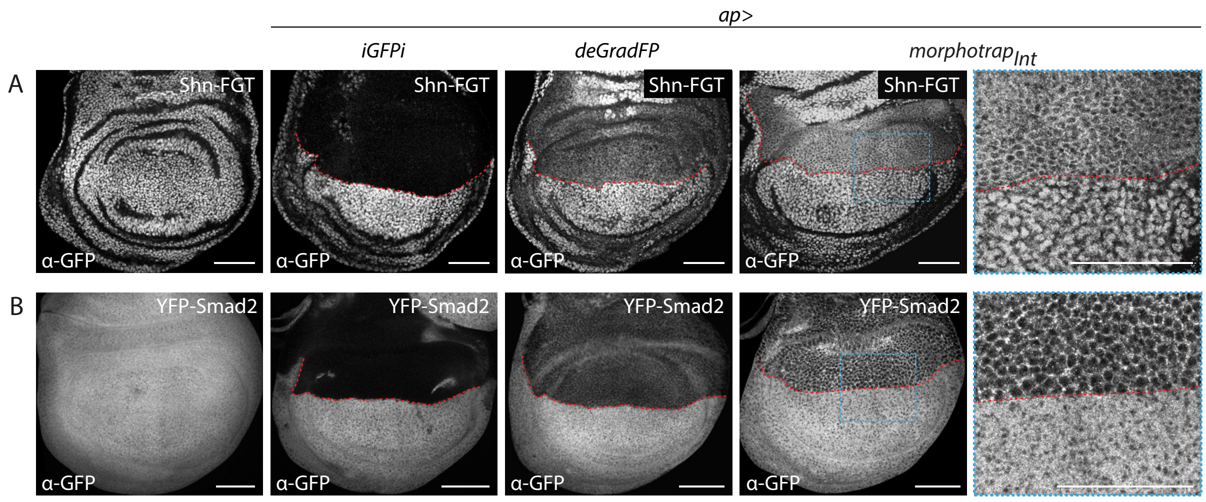


**Fig. S7. Tagged Shn and Smad2 can be manipulated with GFP-based tools.** (A, B) Shn-FGT (A) and YFP-Smad2 (B) visualized by anti-GFP staining in wing discs either expressing the respective tagged allele alone or in combination with the indicated tool in the dorsal compartment using ap-Gal4. Red dashed lines mark dorso-ventral compartment boundary. Scale bars: 50 µm.

**Table S1. Primers to generate plasmids containing homology arms and guide RNAs**

Available for download at
https://journals.biologists.com/dev/article-lookup/doi/10.1242/dev.204222#supplementary-data

**Table S2. Primers to generate reintegration vectors**

Available for download at
https://journals.biologists.com/dev/article-lookup/doi/10.1242/dev.204222#supplementary-data

**Table S3. Primers to generate HA toolbox plasmids**

Available for download at
https://journals.biologists.com/dev/article-lookup/doi/10.1242/dev.204222#supplementary-data

Development • Supplementary information
