# Supplementary material for: A genome-engineered tool set for Drosophila TGF-β/BMP signaling studies
Source: Development. 2024 Nov 18;151(22):dev204222. doi: 10.1242/dev.204222 (PMC11607693; doi:10.1242/dev.204222)
Supplement: Supplementary file 1 [file develop-151-204222_review_history.pdf]

A genome engineered tool set for Drosophila TGF-ß/BMP signaling studies

Clara-Maria Ell, Abu Safyan, Mrinal Chayengia, Manuela M. M. Kustermann, Jennifer Lorenz, Melanie Schächtle and George Pyrowolakis
DOI: 10.1242/dev.204222

Editor: James Briscoe

Review timeline

Original submission:	1 July 2024
Editorial decision:	29 July 2024
First revision received:	1 October 2024
Accepted:	15 October 2024

Original submission

First decision letter

MS ID#: DEVELOP/2024/204222

MS TITLE: A genome engineered tool set for Drosophila TGF-ß/BMP signaling studies

AUTHORS: Clara-Maria Ell; Abu Safyan; Mrinal Chayengia; Manuela M. M. Kustermann; Jennifer Lorenz; Melanie Schächtle; Giorgos Pyrowolakis

I have now received all the referees' reports on the above manuscript, and have reached a decision. The referees' comments are appended below, or you can access them online: please go to:

As you will see, the referees express considerable interest in your work, but have some significant criticisms and recommend a substantial revision of your manuscript before we can consider publication. The referees have several suggestions to help improve your manuscript and clarify specific issues. In addition, Referee 1 raises a specific issue with your Mad isoform and has offered to communicate directly with you to help resolve this issue. I will put you in touch via a separate email.

If you are able to resolve the issue with the Mad allele and revise the manuscript along the lines suggested, I will be happy to receive a revised version of the manuscript. Your revised paper will be re-reviewed by one or more of the original referees, and acceptance of your manuscript will depend on your addressing satisfactorily the reviewers' major concerns. Please also note that Development will normally permit only one round of major revision. If it would be helpful, you are welcome to contact us to discuss your revision in greater detail. Please send us a point-by-point response indicating your plans for addressing the refereesâ€™ comments, and we will look over this and provide further guidance.

Reviewer 1

Advance summary and potential significance to field

© 2024. Published by The Company of Biologists under the terms of the Creative Commons Attribution License (https://creativecommons.org/licenses/by/4.0/).

The manuscript entitled "A genome engineered tool set for Drosophila TGF-ß/BMP signaling studies" describes highly customized editing for all the key genes of the Drosophila TGF-beta network, carefully considering gene structure, transcription, and translation isoforms.
In a first instance, we can envision the tools being useful in several ways:
1. The existing tagged alleles can help researchers easily access spatial expression at the tissue level, subcellular distribution, and differential expression under perturbation conditions when the TGF-beta components are candidate targets of their research.
2. The attP mutant alleles of all 14 genes allow for the easy generation of new alleles for specific research purposes, such as FRT-flanked conditional alleles, alternative tags like Halo tags, and mutations in specific protein domains or intronic regulatory elements.
3. The tagged transcription factors, including Mad, Med, Brk, and Shn, can be used for ChIP-seq assays to understand the genome-wide transcription regulation.

Comments for the author

The manuscript is well-written, with precise descriptions of the genomic editing steps. This clarity will enable future modifications of the generated alleles, making them adaptable for further research. The tools described will benefit not only for researchers studying the TGF-beta signaling pathway. Since many research subjects intersect with this signaling pathway, these tools also be useful to researchers studying process that may be affected by (or modulate) TGF-beta signaling. Therefore, this paper will be of wide interest to Drosophila developmental biologists. It will also provide a template for similar work in other model systems.
We note two results from the manuscript that will need special consideration/clarification:
The first one concerns the discrepancy in expression between the newly generated YFP-Dally and the existing transcription reporter dally-lacZ. The authors propose that this discrepancy may be due to post-transcriptional regulation of the gene or differences in beta-galactosidase and Dally protein stability. Given that the glypican's role in extracellular ligand transportation is still an active area of research, clarifying this aspect seems important. We wonder if the authors couls address this question experimentally and provide a more rigorous explanation.
The second issue arises because the authors' results on Mad isoforms clash with our own (unpublished and hence unbeknownst to the authors). Our lab has generated a GFP-tagged version of the Mad protein, specifically targeting the Mad-PB isoforms. The GFP insertion is precisely at the position of the 3XHA tag presented in the paper (after the second nucleotide of the second exon). However, while the authors observed an absence of Mad-PB expression throughout larval wing disc development, we observed GFP signals throughout wing disc tissues at all larval stages, with prominent nuclear signal near the Dpp source, reflecting the nuclear localization of the phosphorylated Mad protein. Furthermore, in trapping experiments, GFPMad-PB homozygous flies emerged without adult wing tissues (resembling dpp mutants) when co-expressing rotund-Gal4 and UAS-Nb(intracellular)-trap. This conflicts with the manuscript's result where Mad-PB mutant flies show a very minor adult wing phenotype. We have no explanation for the discrepancy, but we think it is important to resolve it before the present manuscript is published. We would be happy to send our line to the authors to facilitate this.
Assuming that the above issues are resolved, this manuscript describes a robust and comprehensive toolset for the fly research community, with precise and reproducible methodologies that will enhance significantly research in TGF-beta signalling and beyond.

Reviewer 2

Advance summary and potential significance to field

The manuscript submited by Pyrowolakis and colleagues describes the generation and use of an toolbox which allows to visualize and manipulate the core components of the Drosophila BMP and Activin pathway. An incredible amount of work was invested to generate a comprehensive tool box of endogenously tagged genes (including 14 genes and several different tags!) and a complmentory set of protein binder-based reagents to manipulate these tagged proteins.
The authors use these fly strains to generate clean loss-of-function alleles and for structure-function analyses. In particular, they test the role of different isoforms of receptors and study structural aspects of Mad and Dally.

© 2024. Published by The Company of Biologists under the terms of the Creative Commons Attribution License (https://creativecommons.org/licenses/by/4.0/).

The manuscript is extremely well written, references to primary literature is abundant and he experiments appaer to be done carefully.

Comments for the author

To my knowledge, this represents the first and the only comprehensive signaling tool box for any signaling pathway. These fly strains will be useful to a large community of researchers and set an standart for future studies on cell signalingI suggest acceptance of the manuscript as is.

Reviewer 3

Advance summary and potential significance to field

Pyrowolakis and colleagues have provided a comprehensive library of endogenously epitope- or fluorescently tagged versions of receptors, co-receptors, transcription factors and feedback regulators generated for the Drosophila BMP and Activin signaling pathways. These engineered alleles are biologically active, allowing for the assessment of protein distribution within tissues and subcellular compartments. Additionally, protein binder-based tools facilitate the visualization and manipulation of epitope-tagged proteins. This resource provides valuable insights for analyzing BMP/Activin signaling. The collection of attP alleles and the library of epitope- and fluorescently tagged BMP/Activin components are expected to greatly assist research in BMP and Activin signaling across different Drosophila tissues and processes. Furthermore, the newly established HA-protein binder tools complement existing collections and will be useful for studies beyond BMP signaling. Therefore, this research provides resources of major value to the developmental biology community and is available to the community with minimal restrictions. I strongly support that this paper will contribute to the community. However, there are several issues that need to be addressed before publications as listed below.

Comments for the author

* Page 7: Regarding the data not shown, if possible, data may be added in the supplemental figures.
* Fig. 2: It would be helpful if the authors interpret the data by adding signaling of BMP (anti-pSmad1/5/8 (pMad)) and Activin (anti-pSmad2/3) of larval wing imaginal discs and egg chamber, along with their schematics throughout the data represented in Figures 2-4.
* The paragraph explaining the pattern distribution of tagged proteins was written in a different order: Fig 2C (Tkv), 2F (Wit), 2E (Put), 2B (Sax), and 2D (Babo). Ensuring consistence in the presentation of the tagged protein sequence would be helpful.
* Fig. 2K: It would be beneficial if the position of the stained cells is indicated in the schematics.
* Page 9: Generating a subtitle for Fig. 3 would be useful.
* Page 10 line 6: Referring to Fig. 3E''', clarification is needed.
* Fig. 4B, C: The authors compared the mid-3rd instar of HA-Mad and HA-Mad-PB. While HA-Mad-PB was absent in Fig. 4B, it appeared in four patches of late larval wing discs in Fig. 4C. Could you clarify how many hours separate mid- and late-wing discs when HA-Mad-PB can appear?
* Fig. 4D, E: There is a noticeable increase in the signal of HA-Mad-PB in the dorsal and ventral compartments in Fig. 4D (HA-Mad-PB, ap>TkvQD) and Fig. 4E (HA-Mad-PB, ap>Tkv-RNAi), respectively, compared to HA-Mad-PB (Fig. 4C). This suggests stronger expression of HA-Mad-PB in late larval wing discs due to BMP manipulation. It would be useful if the authors argue the expression of HA-Mad-PB in the mid-3rd instart in these BMP manipulation cases.
* Fig. 4F: Mad-PB specific phenotypes appear interesting. Analyzing the size of larval wing imaginal discs could provide insights into organ size regulation at different stages.
* Fig. 4F, Fig. 5B, D, E, Fig. S2B: Are the wings derived from females or males? Describing how wings are collected and any differences between female and male wings would be important.
* Fig. 6C: While ap>GrabHAint definitely changes Brk protein localization, BrdU staining may reveal differences in proliferation between dorsal and ventral compartments. How do the authors interpret this data? A more precise analysis and explanation would be preferred.

© 2024. Published by The Company of Biologists under the terms of the Creative Commons Attribution License (https://creativecommons.org/licenses/by/4.0/).

First revision

Author response to reviewers' comments

REVIEWER 1
Reviewer 1: SUMMARY OF THE ADVANCE MADE IN THIS PAPER AND ITS POTENTIAL SIGNIFICANCE TO THE FIELD

The manuscript entitled "A genome engineered tool set for Drosophila TGF-β/BMP signaling studies" describes highly customized editing for all the key genes of the Drosophila TGF-beta network, carefully considering gene structure, transcription, and translation isoforms.
In a first instance, we can envision the tools being useful in several ways:
1. The existing tagged alleles can help researchers easily access spatial expression at the tissue level, subcellular distribution, and differential expression under perturbation conditions when the TGF-beta components are candidate targets of their research.
2. The attP mutant alleles of all 14 genes allow for the easy generation of new alleles for specific research purposes, such as FRT-flanked conditional alleles, alternative tags like Halo tags, and mutations in specific protein domains or intronic regulatory elements.
3. The tagged transcription factors, including Mad, Med, Brk, and Shn, can be used for ChIP-seq assays to understand the genome-wide transcription regulation.

SUGGESTIONS TO AUTHORS

The manuscript is well-written, with precise descriptions of the genomic editing steps. This clarity will enable future modifications of the generated alleles, making them adaptable for further research. The tools described will benefit not only for researchers studying the TGF-beta signaling pathway. Since many research subjects intersect with this signaling pathway, these tools also be useful to researchers studying process that may be affected by (or modulate) TGF-beta signaling. Therefore, this paper will be of wide interest to Drosophila developmental biologists. It will also provide a template for similar work in other model systems.

Thanks a lot!

We note two results from the manuscript that will need special consideration/clarification: The first one concerns the discrepancy in expression between the newly generated YFP- Dally and the existing transcription reporter dally-lacZ. The authors propose that this discrepancy may be due to post-transcriptional regulation of the gene or differences in beta-galactosidase and Dally protein stability. Given that the glypican's role in extracellular ligand transportation is still an active area of research, clarifying this aspect seems important. We wonder if the authors couls address this question experimentally and provide a more rigorous explanation.

The statement on the discrepancy between reporter-based expression patterns and expression pattern of the tagged alleles concerns our observations of YFP-Dally expression in the germarium. Previous studies have (i) identified *dally* expression in cap cells of the germarium via a *dally-lacZ* reporter and (ii) have solidly established a role of Dally in the same cells in Dpp-dependent germline stem cell maintenance. Despite the fact that our YFP-Dally allele is biologically active, we experienced difficulties to reliably and robustly detect YFP-Dally in cap cells in the germarium, a tissue that is prone (at least in our hands) to produce quite high background and antibody precipitates in immunohistochemistry. In the meanwhile, we optimized our staining protocol and used different antibodies that enabled eventually detecting robust but low expression of YFP- Dally in somatic cap cells. With these new results in hindsight (which are now included in the revised fig S5), we have relativized the original statement as the differences to reporter expression are restricted to quantitative aspects only and probably stem from the trivial fact that the dynamics of a membrane-bound signaling protein (YFP-Dally) cannot be compared to nuclear β-Galactosidase expressed from a reporter construct.

© 2024. Published by The Company of Biologists under the terms of the Creative Commons Attribution License (https://creativecommons.org/licenses/by/4.0/).

The second issue arises because the authors' results on Mad isoforms clash with our own (unpublished and hence unbeknownst to the authors). Our lab has generated a GFP-tagged version of the Mad protein, specifically targeting the Mad-PB isoforms. The GFP insertion is precisely at the position of the 3XHA tag presented in the paper (after the second nucleotide of the second exon). However, while the authors observed an absence of Mad-PB expression throughout larval wing disc development, we observed GFP signals throughout wing disc tissues at all larval stages, with prominent nuclear signal near the Dpp source, reflecting the nuclear localization of the phosphorylated Mad protein. Furthermore, in trapping experiments, GFPMad-PB homozygous flies emerged without adult wing tissues (resembling dpp mutants) when co-expressing rotund-Gal4 and UAS-Nb(intracellular)-trap. This conflicts with the manuscript's result where Mad-PB mutant flies show a very minor adult wing phenotype. We have no explanation for the discrepancy, but we think it is important to resolve it before the present manuscript is published. We would be happy to send our line to the authors to facilitate this.

We thank the reviewer for the remark and the editor for establishing contact with the reviewer (JP Vincent). We had extensive discussions with the lab of JP Vincent, including two zoom calls on this issue over the last two months. By cross-checking our experimental strategies, we concluded that our constructs are nearly identical, despite showing different expression patterns. We have no explanation for the difference. Since our phenotypic analysis is more extensive than that of the Vincent lab, we have agreed with JP Vincent that we should report our construct as is.

REVIEWER 2

Reviewer 2: SUMMARY OF THE ADVANCE MADE IN THIS PAPER AND ITS POTENTIAL SIGNIFICANCE TO THE FIELD

The manuscript submited by Pyrowolakis and colleagues describes the generation and use of an toolbox which allows to visualize and manipulate the core components of the Drosophila BMP and Activin pathway. An incredible amount of work was invested to generate a comprehensive tool box of endogenously tagged genes (including 14 genes and several different tags!) and a complmentory set of protein binder-based reagents to manipulate these tagged proteins.
The authors use these fly strains to generate clean loss-of-function alleles and for structure-function analyses. In particular, they test the role of different isoforms of receptors and study structural aspects of Mad and Dally.
The manuscript is extremely well written, references to primary literature is abundant and he experiments appaer to be done carefully.

SUGGESTIONS TO AUTHORS
To my knowledge, this represents the first and the only comprehensive signaling tool box for any signaling pathway. These fly strains will be useful to a large community of researchers and set an standart for future studies on cell signalingI suggest acceptance of the manuscript as is.

Thank you very much!

REVIEWER 3

Reviewer 3: SUMMARY OF THE ADVANCE MADE IN THIS PAPER AND ITS POTENTIAL SIGNIFICANCE TO THE FIELD

Pyrowolakis and colleagues have provided a comprehensive library of endogenously epitope- or fluorescently tagged versions of receptors, co-receptors, transcription factors and feedback regulators generated for the Drosophila BMP and Activin signaling pathways. These engineered alleles are biologically active, allowing for the assessment of protein distribution within tissues and subcellular compartments. Additionally, protein binder-based tools facilitate the visualization and manipulation of epitope-tagged proteins. This resource provides valuable insights for analyzing BMP/Activin signaling. The collection of attP alleles and the library of epitope- and fluorescently tagged BMP/Activin components are expected to greatly assist research in BMP and Activin signaling across different Drosophila tissues and processes. Furthermore, the newly established HA-protein binder tools complement existing collections and will be useful for studies beyond BMP signaling.

© 2024. Published by The Company of Biologists under the terms of the Creative Commons Attribution License (https://creativecommons.org/licenses/by/4.0/).

Therefore, this research provides resources of major value to the developmental biology community and is available to the community with minimal restrictions. I strongly support that this paper will contribute to the community. However, there are several issues that need to be addressed before publications as listed below.

Thank you very much for your time and suggestions!
During the revision process, we have addressed all issues raised. In the following lines, we present our actions to the individual points.

SUGGESTIONS TO AUTHORS

* Page 7: Regarding the data not shown, if possible, data may be added in the supplemental figures.

We generated a new supplementary figure (Fig. S3) to include the missing data. We illustrate for the receptors and glypicans that the observed signals are specific: The epitope immunoreactivity can be eliminated via compartment-specific expression of the corresponding RNAi constructs.

* Fig. 2: It would be helpful if the authors interpret the data by adding signaling of BMP (anti-pSmad1/5/8 (pMad)) and Activin (anti-pSmad2/3) of larval wing imaginal discs and egg chamber, along with their schematics throughout the data represented in Figures 2- 4.

Thank you for the suggestion. We now expanded Fig. 2 to include the activity pattern of BMP signaling in the wing imaginal disc (Fig. 2B), during egg chamber development (Fig. 2K,K') and in the germline stem cell niche (Fig. 2L). To our knowledge, there are no tools available to reliably monitor Activin signaling activity in *Drosophila*.

* The paragraph explaining the pattern distribution of tagged proteins was written in a different order: Fig 2C (Tkv), 2F (Wit), 2E (Put), 2B (Sax), and 2D (Babo). Ensuring consistence in the presentation of the tagged protein sequence would be helpful.

We have now expanded and restructured Fig. 2 (see also previous point). We changed the order of the panels to align with the paragraph in the main text body.

* Fig. 2K: It would be beneficial if the position of the stained cells is indicated in the schematics.

Thanks for the suggestion. We have now added a box in the schematic of Fig. 2J indicating the area that is depicted in the stainings of Fig. 2L,N and Fig. S5B,D,F,H,J.

* Page 9: Generating a subtitle for Fig. 3 would be useful.

We assume the reviewer suggests to split the rather long paragraph with the title “Assessing tissue and cellular distribution of tagged BMP components” into two parts: one including the plasma membrane components (receptors, glypicans) with the contents of Fig. 2 and a second on the intracellular components (transcription factors, Fig. 3). We have changed this accordingly.

* Page 10 line 6: Referring to Fig. 3E''', clarification is needed.

Thank you, this is now corrected.

* Fig. 4B, C: The authors compared the mid-3rd instar of HA-Mad and HA-Mad-PB. While HA-Mad-PB was absent in Fig. 4B, it appeared in four patches of late larval wing discs in Fig. 4C. Could you clarify how many hours separate mid- and late-wing discs when HA-Mad-PB can appear?

We have now included new data with staged larvae and quantifications that describe the emergence of HA-Mad-PB during late stages of third instar larval disc development (Fig. S6D). The pattern appears indeed very late in larval development and is fully present at the transition into pupal development.

* Fig. 4D, E: There is a noticeable increase in the signal of HA-Mad-PB in the dorsal and ventral

© 2024. Published by The Company of Biologists under the terms of the Creative Commons Attribution License (https://creativecommons.org/licenses/by/4.0/).

compartments in Fig. 4D (HA-Mad-PB, ap>TkvQD) and Fig. 4E (HA-Mad-PB, ap>Tkv-RNAi), respectively, compared to HA-Mad-PB (Fig. 4C). This suggests stronger expression of HA-Mad-PB in late larval wing discs due to BMP manipulation. It would be useful if the authors argue the expression of HA-Mad-PB in the mid-3rd instart in these BMP manipulation cases.

If we understand correctly, this question is connected to the one above. Our results indeed make the point that BMP signaling activates Mad-PB: Reduction of BMP signaling in the dorsal compartment (*ap>Tkv-RNAi*) reduces Mad-PB - as a side effect of this manipulation the ventral expression increases because dorsal depletion of Tkv results in increase of ligand levels that are flooding into the ventral compartment. In reverse, activation of BMP in the dorsal compartment (*ap>Tkv^{QD}*) increases Mad-PB. All effects (reduction and increase of Mad-PB) are compared to wild-type levels as control. We looked more carefully into the onset of expression of HA-Mad-PB upon increase of BMP signaling (as suggested by the reviewer) and found no premature Mad-PB expression. The findings, which are now included in a new panel (Fig. S6E), suggest that while BMP signaling is required for Mad-PB activation and controls its spatial extend, the onset of expression is gated by other factors.

* Fig. 4F: Mad-PB specific phenotypes appear interesting. Analyzing the size of larval wing imaginal discs could provide insights into organ size regulation at different stages.

Indeed, the Mad-PB specific effects on adult wing size are reproducible but subtle (see Fig. 4F). This, along with the fact that Mad-PB is activated only very late in larval development, suggests that monitoring sizes of wing discs during larval development might not be conclusive. An analysis of pupal requirements of Mad-PB would probably be more adequate but are, we believe, outside of the scope of the present study.

* Fig. 4F, Fig. 5B, D, E, Fig. S2B: Are the wings derived from females or males? Describing how wings are collected and any differences between female and male wings would be important.

Thank you for the remark. We corrected this omission by adding the information in the figure legends and the methods section.

* Fig. 6C: While ap>GrabHAint definitely changes Brk protein localization, BrdU staining may reveal differences in proliferation between dorsal and ventral compartments. How do the authors interpret this data? A more precise analysis and explanation would be preferred.

Brk expression is restricted to lateral cells of the wing disc by direct BMP-dependent transcriptional repression. The protein localizes to the nucleus and negatively affects cell proliferation: We show that the GrabHAInt can efficiently affect Brk at two levels: cellular localization and - connected to that – function. We show that in discs expressing GrabHAInt in the dorsal compartment, HA-Brk is not present anymore in the nucleus but rather accumulates at cell membranes. At the same time, this results in an increase in lateral proliferation rates in the manipulated compartment (BrdU staining). In addition, HA-Brk depletion from the nucleus throughout the wing pouch (*nub*>GrabHAInt) also affects adult wing shape and size. Both effects can be “phenocopied” using iGFPi on YFP-Brk and are consistent with previous studies using *brk* hypomorphic alleles (Campbell and Tomlinson, 1999; Schwank et al., 2008) or RNAi-mediated *brk* depletion (Barrio and Milán, 2017). We have reformulated this section for more clarity.

Second decision letter

MS ID#: DEVELOP/2024/204222

MS TITLE:A genome engineered tool set for Drosophila TGF-β/BMP signaling studies

AUTHORS: Clara-Maria Ell; Abu Safyan; Mrinal Chayengia; Manuela M. M. Kustermann; Jennifer Lorenz; Melanie Schächtle; Giorgos Pyrowolakis
ARTICLE TYPE: Techniques and Resources Article

© 2024. Published by The Company of Biologists under the terms of the Creative Commons Attribution License (https://creativecommons.org/licenses/by/4.0/).

I am happy to tell you that your manuscript has been accepted for publication in Development, pending our standard publication integrity checks.

Reviewer 1

Advance summary and potential significance to field

See previous review

Comments for the author

Our opinion of the manuscript by Ell et al remains positive. In light of the importance of the Dpp pathway, the strains reported will be of interest to many Drosophila biologists.
The authors have addressed our question about the expression of YFP-Dally in the germarium. With respect to the different behaviour of our Mad knock-in and that reported by the paper, I confirm the extensive discussions between our two labs. We agree that the two constructs are nearly identical. The differences in expression cannot be explained, but the onus will be on my group to explain it (in due course) since, the present paper has priority and is based on more extensive characterisation so far.

Reviewer 2

Advance summary and potential significance to field

not requested

Comments for the author

None

Reviewer 3

All my previous concerns have been thoroughly addressed. Therefore, I support this paper for publication.

© 2024. Published by The Company of Biologists under the terms of the Creative Commons Attribution License (https://creativecommons.org/licenses/by/4.0/).
